# Increased neutrophil extracellular traps promote metastasis potential of hepatocellular carcinoma via provoking tumorous inflammatory response

**DOI:** 10.1186/s13045-019-0836-0

**Published:** 2020-01-06

**Authors:** Lu-Yu Yang, Qin Luo, Lu Lu, Wen-Wei Zhu, Hao-Ting Sun, Ran Wei, Zhi-Fei Lin, Xiang-Yu Wang, Chao-Qun Wang, Ming Lu, Hu-Liang Jia, Jin-Hong Chen, Ju-Bo Zhang, Lun-Xiu Qin

**Affiliations:** 1 Department of General Surgery, Huashan Hospital, Fudan University, 12 Urumqi Road (M), Shanghai, 200040 China; 2 Cancer Metastasis Institute, Fudan University, Shanghai, China; 3 Institute of Biomedical Sciences, Fudan University, Shanghai, China; 4 Department of Infection Disease, Huashan Hospital, Fudan University, 12 Urumqi Road (M), Shanghai, 200040 China

**Keywords:** NETs, Hepatocellular carcinoma metastasis, Inflammatory response, COX2, Translation

## Abstract

**Background:**

The propensity of the activated neutrophils to form extracellular traps (NETs) is demonstrated in multiple inflammatory conditions. In this study, we investigated the roles of NETs in metastasis of hepatocellular carcinoma (HCC) and further explored the underlying mechanism of how NETs affect metastasis as well as the therapeutic value.

**Methods:**

The neutrophils were isolated from the blood of human HCC patients and used to evaluate the formation of NETs. The expression of NET markers was detected in tumor specimens. A LPS-induced NET model was used to investigate the role of NETs on HCC metastasis. RNA-seq was performed to identify the key molecular event triggered by NETs, and their underlying mechanism and therapeutic significance were explored using both in vitro and in vivo assays.

**Results:**

NET formation was enhanced in neutrophils derived from HCC patients, especially those with metastatic HCCs. NETs trapped HCC cells and subsequently induced cell-death resistance and enhanced invasiveness to trigger their metastatic potential, which was mediated by internalization of NETs into trapped HCC cells and activation of Toll-like receptors TLR4/9-COX2 signaling. Inhibition of TLR4/9-COX2 signaling abrogated the NET-aroused metastatic potential. A combination of DNase 1 directly wrecking NETs with anti-inflammation drugs aspirin/hydroxychloroquine effectively reduced HCC metastasis in mice model.

**Conclusions:**

NETs trigger tumorous inflammatory response and fuel HCC metastasis. Targeting NETs rather than neutrophils themselves can be a practice strategy against HCC metastasis.

## Background

Inflammatory activation, an interaction between host and cancer cell, is often triggered in cancer development and metastasis [1, 2]. Meanwhile, anti-inflammation drugs have displayed certain anti-metastasis effects through less clarified mechanism [3]. Neutrophils are the most abundant immune cells and have a fundamental role in inflammatory responses, but their contribution to metastasis is still controversial [4–7]. It is reported that tumor-entrained neutrophils may induce an inhibitory process at the metastatic site [8]. However, more studies indicate that neutrophil recruitment to the pre-metastatic site play critical roles in the metastatic initiation [9].

Neutrophil extracellular traps (NETs) are extensive released extracellular web-like structures that are composed of cytosolic protein assembled on a scaffold of released chromatin from the activated neutrophils [10]. They are originally discovered as the innate immune defensive process to trap and kill invading pathogens and then found wildly associated with various pathological conditions including autoimmune response, clot-related disorders, wound healing, organ impairment, and sterile inflammation [11, 12]. The recognition of NETs in cancer is just emerging. Links have been made between NETs and metastasis in some mice models, and evidences of NETs’ presence have been reported in some certain tumors [13–16]. Despite these, how NETs affect metastasis remains to be explored.

Hepatocellular carcinoma (HCC) accounts for the second leading cause of cancer-related death. The most frequent metastasis destinations of HCC are the liver and lung [17]. High infiltration of tumor-associated neutrophils and elevated neutrophil-lymphocyte ratio (NLR) has been observed in HCC, which were correlated with worse outcome [18–20]. But little is known about the specific role of neutrophils, especially NETs, on HCC metastasis. Thus, we designed this study to reveal participation of NETs in HCC metastasis and uncover the mechanism of NETs’ role on metastasis cascade. Moreover, we studied the inflammatory response triggered by NETs and combined breaking NETs plus anti-inflammation therapies against HCC metastasis.

## Methods

### Human specimens, animal models, and cell lines

Surgical samples or peripheral blood were obtained from HCC patients or healthy donors (HD) in our institute. Six to 8 weeks old of C57BL/6 male mice or null mice were used in animal studies. The human cell line HepG2 and L02 were obtained from Chinese Academy of Sciences. The human cell line MHCC97H and mice cell line Hepa1-6 were obtained from the Liver Cancer Institute, Fudan University. Detail information is described in Additional file 1.

### NET formation study

To evaluate NET formation capacity, freshly isolated human or mice neutrophils were adjusted to a concentration of 5 × 10^5^ cells/ml and stimulated with Phorbol 12-myristate 13-acetate (PMA, 20 nM, Sigma-Aldrich) for indicated hours, with or without DNase 1(100 U/ml, Sigma-Aldrich) to allow NET formation. In CM-induced and plasma-induced NET formation assay, neutrophils were incubated with corresponding CM (1:2) for 30 min before PMA stimulation or incubated in plasma of HCC patients or HD. For co-culture, 1 × 10^5^ indicated HCC cells were seeded on upper chamber, 5 × 10^5^ species-matched normal neutrophils were seeded on lower chamber of 8-μM Transwell system for 16–20 h incubation. In LPS-induced NET model, isolated neutrophils were directly incubated for 4 h to form NETs. In some assays, the neutrophils were pretreated with hydroxychloroquine (HCQ, 50 μM, R&D) and Aspirin (5 mM, Sigma-Aldrich) for 30 min before CM administration to inhibit CM-induced NET formation.

For visualization, neutrophils were seeded on 96-well plates for corresponding incubation, and cell-impermeable DNA dye SytoxGreen (Thermo Fisher Scientific, 1:10000) and cell-permeable DNA dye Hoechst33342 (Thermo Fisher Scientific, 1:1000) were added to the incubation system. At the end of incubation, the plates were directly moved to fluorescence microscope (Leica) for NET formation visualization. In some cases, neutrophils were seeded on coverslips in 24-well plates to generate NETs as described above, and then, the formed NETs were fixed for further immunofluorescence detection.

For quantification, NET DNA generated by neutrophils was digested with 500 mU/ml micrococcal nuclease (MNase). The nuclease activity was stopped with Ethylenediaminetetraacetic acid (EDTA, 5 mM), and the culture supernatants were collected and stored at – 80 °C until further use. NET DNA in the supernatants was quantified by PicoGreen® dsDNA Quantitation Reagent (Thermo Fisher Scientific) with fluorescence spectrometry under filter setting of 480 nm/520 nm excitation/emission and semi-quantitatively standardized to control group.

### Preparation of NETs

Neutrophils were isolated and seeded on 6-well plates (1 × 10^7^/well). Human neutrophils were stimulated with PMA (20 nM) for 4 h, and neutrophils from LPS-treated C57BL/6 mice were incubated in medium for 4 h to form NETs. Then, the supernatants were discharged carefully by slow suction and washed twice to eliminate residual PMA or NET-unassociated substances without disturbing NETs. RPMI (1 mL) containing MNase (1 U/mL) was then added to digest NETs at 37 °C for 20 min followed by 5 mM EDTA to stop nuclease activity. The supernatant containing NETs was collected and centrifuged to eliminate cell debris. Isolated NETs were stored at − 80 °C for further use.

### Measurement of serum MPO-DNA level

We measured MPO-DNA complexes in human and mice serum using a well-adopted capture ELISA assay with some modification [21]. Briefly, as the capturing antibody, 5 μg/mL anti-MPO monoclonal antibody was coated to 96-well plates overnight at 4 °C. After blocking in 1% BSA, 100 μl of diluted serum was added per well and incubated at room temperature on a shaking device for 2 h. After washing five times with PBST, PicoGreen® dsDNA Quantitation Reagent was added according to manufacturer’s directions. The values were then read with a fluorometer with a filter setting of 480 nm/520 nm excitation/emission and semi-quantitatively standardized to healthy donor or control group.

### Mice model: LPS-induced NET model

We adopted the well-used lipopolysaccharide (LPS)-induced NET model [14, 22]. Briefly, LPS (Sigma, 10 ug/mouse) was intraperitoneally injected to induce systemic inflammation in C57BL/6 mice. DNase 1 (100 U/mouse) was given intraperitoneally daily as abrogation 24 h prior to LPS. A neutrophil-depleting antibody (rat anti-Ly6G; clone 1A8 from BioXcell; 12.5 μg/mouse, intravenously) was given 24 h prior to LPS to deplete neutrophils. To verify NET formation in the inflammation model, mice were sacrificed in 6 h after LPS injection, serum was then collected for MPO-DNA level detection, and peripheral neutrophils were isolated for NET formation assay or generate NETs. The liver and lung were removed to prepare single-cell suspension for neutrophil infiltration detection using flow cytometry. The lung was also fixed by tracheal perfusion with 4% paraformaldehyde (PFA) for 15 min and embedded in O.C.T. compound for frozen sections and subsequent in situ immunofluorescence staining of NETs.

### Mice model: establishment of experimental metastasis in LPS-induced NET model

In 6 h after establishment of the LPS-induced inflammation model in C57BL/6 mice, 2 × 10^6^ Hepa1-6 cells were injected through the tail vein or spleen. DNase 1 (100 U/mouse) abrogation was then given daily. The mice were sacrificed, and intrahepatic/lung metastasis burden was assessed in 20 days. Experimental intrahepatic metastasis burden was assessed by calculating the percentage of hepatic tissue replaced by tumor (the hepatic replacement area, HRA). The lung metastatic lesions were directly counted on tissue sections using H&E staining.

### In vitro assays on invasion, death rate, adhesion, and proliferation of HCC cells

Detail of invasion, death rate, adhesion, and proliferation assay of HCC cells under NET stimulation were described in Additional file 1.

### Statistical analysis

The results are expressed as the means ± SEM. The statistical significance of differences between groups was determined by Student’s *t* tests. Pearson correlation test was used for correlation analysis. Kaplan-Meier method and log-rank test were used for follow-up data. GraphPad statistical software (version 5.0) was used for all statistical analyses. All data were analyzed using two-tailed tests unless otherwise specified, and *P* < 0.05 was considered statistically significant.

Further details of materials and methods are described in Additional files 1 and 3.

## Results

### NET formation is enhanced in neutrophils from patients with HCC, especially metastatic HCC

**Fig. 1:**
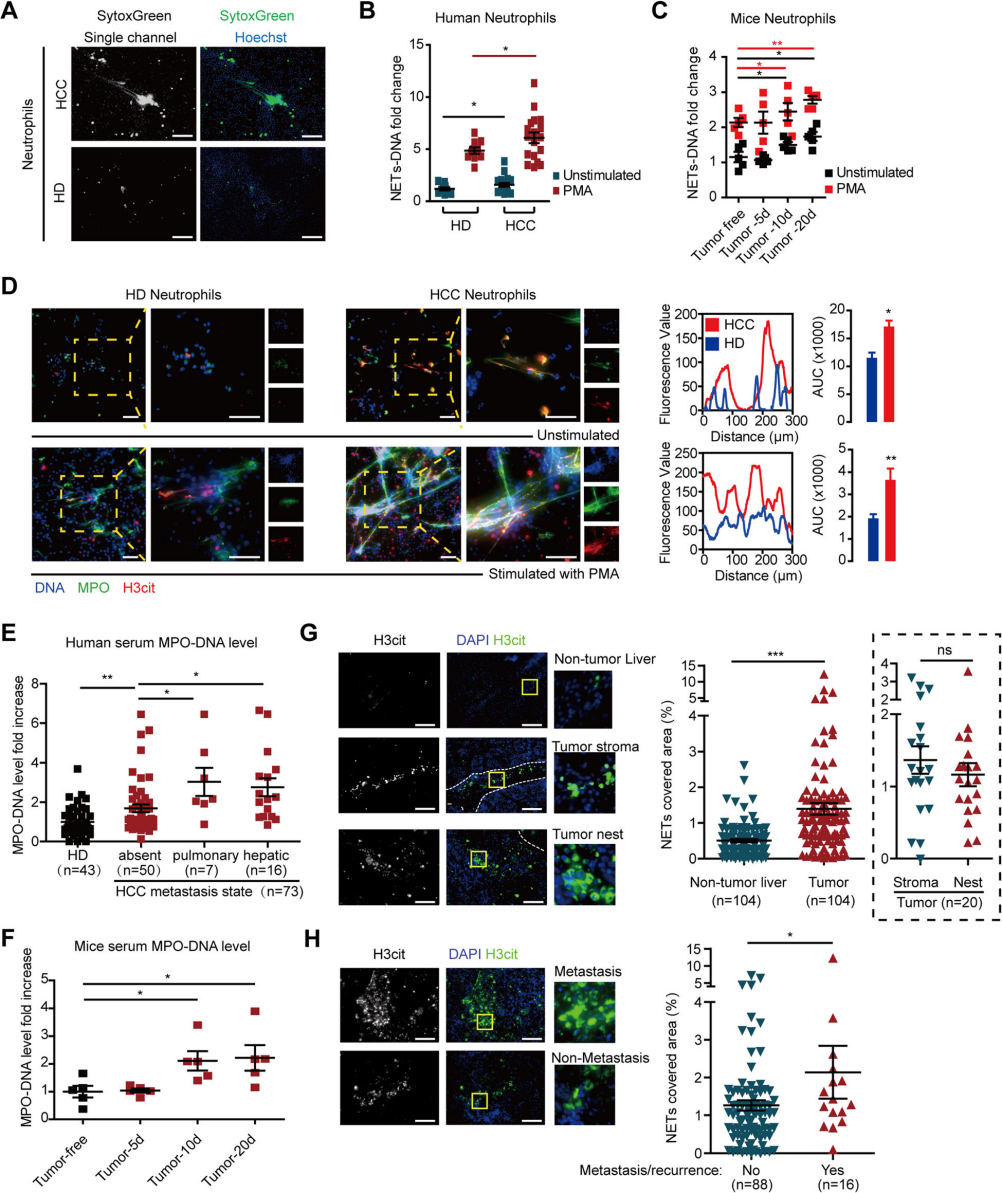
NET formation was enhanced in HCC associated neutrophils. **a** Spontaneous extracellular DNA released from neutrophils HD and HCC patients. Freshly isolated neutrophils were incubated with Hoechst33342 and extracellular DNA dye SytoxGreen for 4 h. Representative images were presented. Scare bar: 100 μm. **b**, **c** Quantification of NET-DNA released from unstimulated or PMA-stimulated neutrophils from human (**b**) or Hepa1-6 orthotopically implanted C57BL/6 mice (**c**) after 4 (human) or 16 (mice) hours incubation. **d** Fluorescent immunostaining of NETs component DNA/MPO/H3cit in unstimulated or PMA-stimulated human neutrophils. Scale bars: 50 μm. The fluorescence value was analyzed utilizing ImageJ. **e** MPO-DNA level in serum samples from HD (*n* = 43) and HCC patients with different metastasis state (*n* = 73). **f** MPO-DNA level in serum samples from Hepa1-6 orthotopically implanted C57BL/6 mice at indicated time point (*n* = 6 each). **g**, **h** Representative images and analysis of NET regional distribution (**g**) and expression dissimilarity with metastasis/recurrence state (**h**) in HCC tissue samples (*n* = 104). Scare bar: 100 μm. *P < 0.05; **P < 0.01; ***P < 0.001; ns, no significance. Data were presented as means ± SEM

Freshly isolated neutrophils were stained with cell-impermeable chromatin dye SytoxGreen to analyze potential NET release. We observed that the neutrophils from HCC patients exhibited an increased capacity of releasing more DNA to extracellular space compared with those from healthy donor (HD) (Fig. 1a). This was further validated using quantification assays in both human and mice (Fig. 1b, c). A significant proportion of neutrophils from HCC patients was in a ready state to form NETs (a pro-NETotic state) with high nuclear expression of H3cit, a citrullinated modification of histone 3 as a featured marker of NETs formation (Additional file 2: Figure S1A), which was further supported by a higher H3cit expression in the neutrophils lysate (Additional file 2: Figure S1B-C). Typical spontaneous NET formation, including morphology transition from delobulated nuclear to spreading extracellular DNA which was decorated with MPO/NE/H3cit, was observed within neutrophils from HCC patients rather than those from HD (Additional file 2: Figures S1A). The enhanced NET formation capacity was further sustained by PMA stimulation, in which a hyper-responsive increase in NET formation was observed presenting a wider range of web-like structure (visualization in Fig. 1d and Additional file 2: Figure S1D with quantification in Fig. 1b, c).

The enhanced spontaneous NETs of HCC neutrophils in the absence of specific stimulation in vitro suggested that NETs already proceeded in HCC patients. As a proof, serum MPO-DNA level, a NET product, was increased, especially in those with metastatic HCCs (Fig. 1e, f). We further sought the evidence of NET formation in HCC tissue samples. A remarkable recruitment of neutrophils into tumor was found (Additional file 2: Figure S2A). The co-localization of NET components MPO- and H3cit-positive neutrophils suggested NET formation from the recruited neutrophils (Additional file 2: Figure S2B-C). NETs (marked by H3cit) were mainly detected in tumor rather than the non-tumor liver tissues, with no significant discrepancy between tumor stroma and nest (Fig. 1g and Additional file 2: Figure S3). Of note, much more NETs were found in metastatic HCC tissues compared with those with metastasis-free (Fig. 1h), but no significant difference was observed in the distribution of neutrophils themselves (Additional file 2: Figure S2A). Moreover, we also found a close correlation between MPO as well as CD66b and PADI4 (protein-arginine deiminase type-4, an essential enzyme to trigger citrullination of histone and initiate NETs) in two databases of TCGA and The Human Protein Atlas (Additional file 2: Figure S4), which further supported our finding of NETs forming in HCC. Taken together, these data suggested neutrophils derived from HCC patients had a higher potential to form NETs, and the enhanced formation of NETs was correlated with metastasis of HCC.

### HCC cells prime normal neutrophils to form NETs

Plasma from HCC patients rather than HD triggered robust NET formation of normal neutrophils (Additional file 2: Figure S5A). Likewise, NETs were also aroused when species-matched normal neutrophils were co-cultured with human HepG2 and MHCC97H cancer cells or mice Hepa1-6 HCC cells, but not with normal cell line L02 (Additional file 2: Figures S5B and S6A). Furthermore, pretreatment of normal neutrophils with conditioned medium of HCC cells (HCC-CM), especially the highly metastatic MHCC97H, also enhanced the NET formation followed by secondary PMA stimulation (Additional file 2: Figures S5C and S6B). Moreover, HCC-CM alone could induce NET formation with prolonged incubation time (Additional file 2: Figure S5D). However, CM from L02 showed no NET-arousing capacity (Additional file 2: Figure S5D). Taken together, these findings demonstrated that HCC cells, especially those with high metastatic potential, could induce normal neutrophils to form NETs via some secreted factor(s).

### NETs promote the experimental metastasis of HCC

**Fig. 2:**
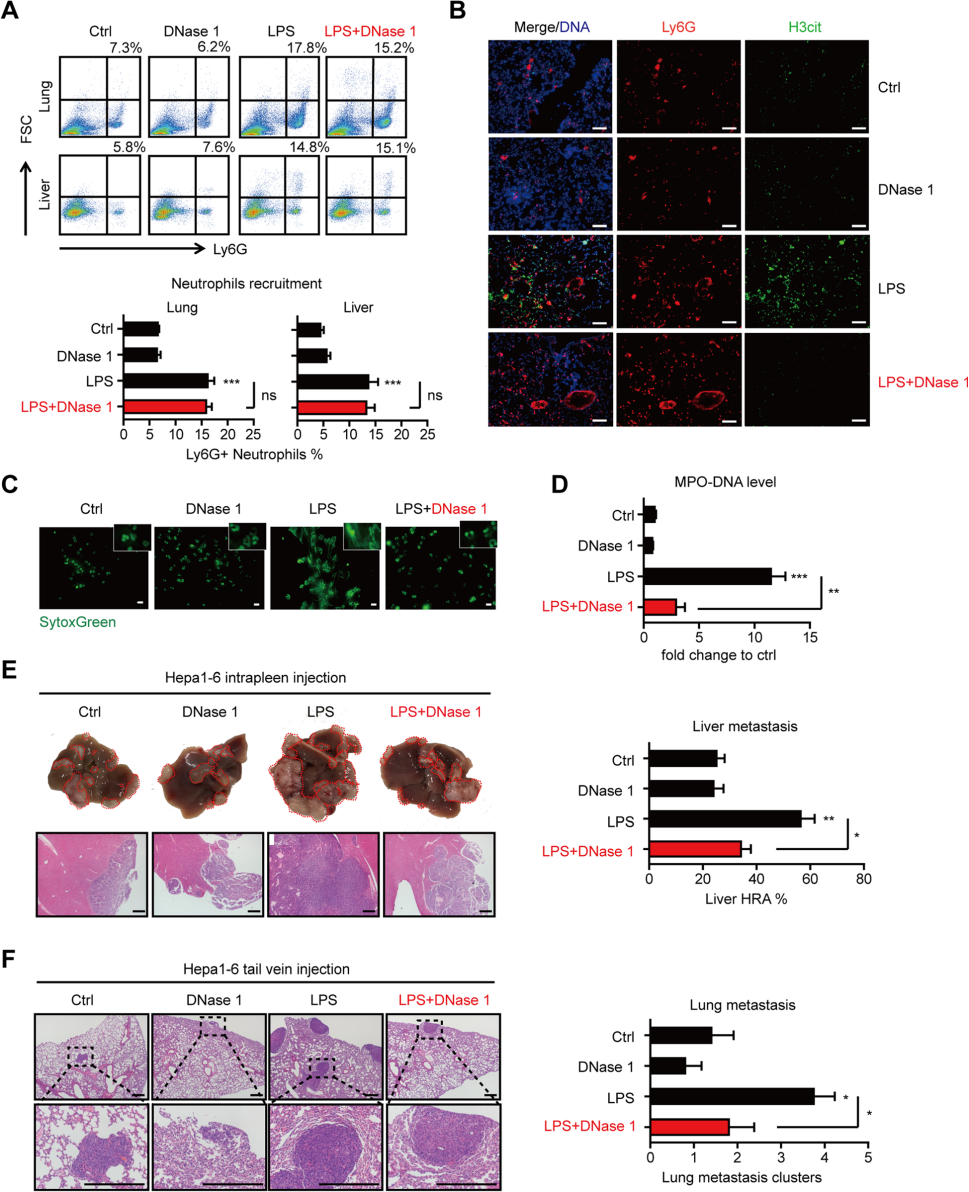
NETs fueled HCC experimental metastasis in a LPS-induced NET model. **a** LPS mobilized neutrophils, and this was not disturbed by DNase 1 in C57BL/6 mice (*n* = 5 each). The liver and lung single-cell suspension was analyzed for neutrophils (Ly6G) infiltration by flow cytometry. Representative plot (upper panel) and quantification (lower panel) were shown. **b** Immunofluorescence detection of DNA, H3cit, and Ly6G revealed NET formation of infiltrated neutrophils in situ in the LPS-induced NET model. Representative images of NETs in the lung were shown. Scare bar: 50 μm. **c** NETs were released by neutrophils from LPS-treated mice, and this was efficiently disrupted by DNase 1 in vitro. Neutrophils were isolated from saline/LPS-treated mice and incubated with/without DNase 1 for 4 h. Neutrophils were then fixed and stained for SytoxGreen to observe NETs. Scare bar: 20 μm. **d** Serum MPO-DNA level was elevated by systemic LPS administration and significantly reduced by DNase 1 abrogation in vivo. **e**, **f** Representative images of the liver (**e**) or lung (**f**) experimental metastasis and quantification in LPS-induced NET model (*n* = 5 each). Hepa1-6 cells were intraspleen or intravenous adopted after LPS and/or DNase 1 administration in C57BL/6 mice. Scare bar: 200 μm. Saline served as a control. *P < 0.05; **P < 0.01; ***P < 0.001. Data were presented as means ± SEM

Next, we sought to investigate the important roles of NETs in HCC metastasis. We induced NETs in immune-component C57BL/6 mice by LPS injection, a model proven efficient in NET induction [14, 22]. LPS resulted in a rapid accumulation of neutrophils in the liver and lung (Fig. 2a), as well as the further NET formation in situ by immunofluorescence staining of neutrophil marker Ly6G and H3cit (Fig. 2b). Isolated neutrophils from LPS-treated mice also underwent significant NET formation in vitro (Fig. 2c). LPS treatment induced an elevation of serum MPO-DNA complex, which further indicated NET formation in vivo (Fig. 2d). DNase 1, an enzyme used to digest extracellular chromatin, effectively digest both in vitro and in vivo formed NETs (Fig. 2c, d), showing comparable efficiency of blocking NETs by depleting neutrophils with little influence on neutrophils recruitment (Fig. 2a and Additional file 2: Figure S7).

We then established experimental metastasis model by intraspleen/intravascular injection of Hepa1-6 cells in the LPS-induced NET model, and significant increased metastases were found in the liver and lung (Fig. 2e, f). Considering that LPS treatment also comprised a series of physiological and pathological response other than NETs, we adopted DNase 1 to digest the formed NETs and found this increased metastasis burden was abrogated, which demonstrated the critical role of NETs as an important inflammatory condition to promote metastasis (Fig. 2e, f). These suggested that NETs played important roles in facilitating HCC metastasis.

### NETs enhanced the cytotoxicity resistance and invasion capacity of the trapped HCC cells

**Fig. 3:**
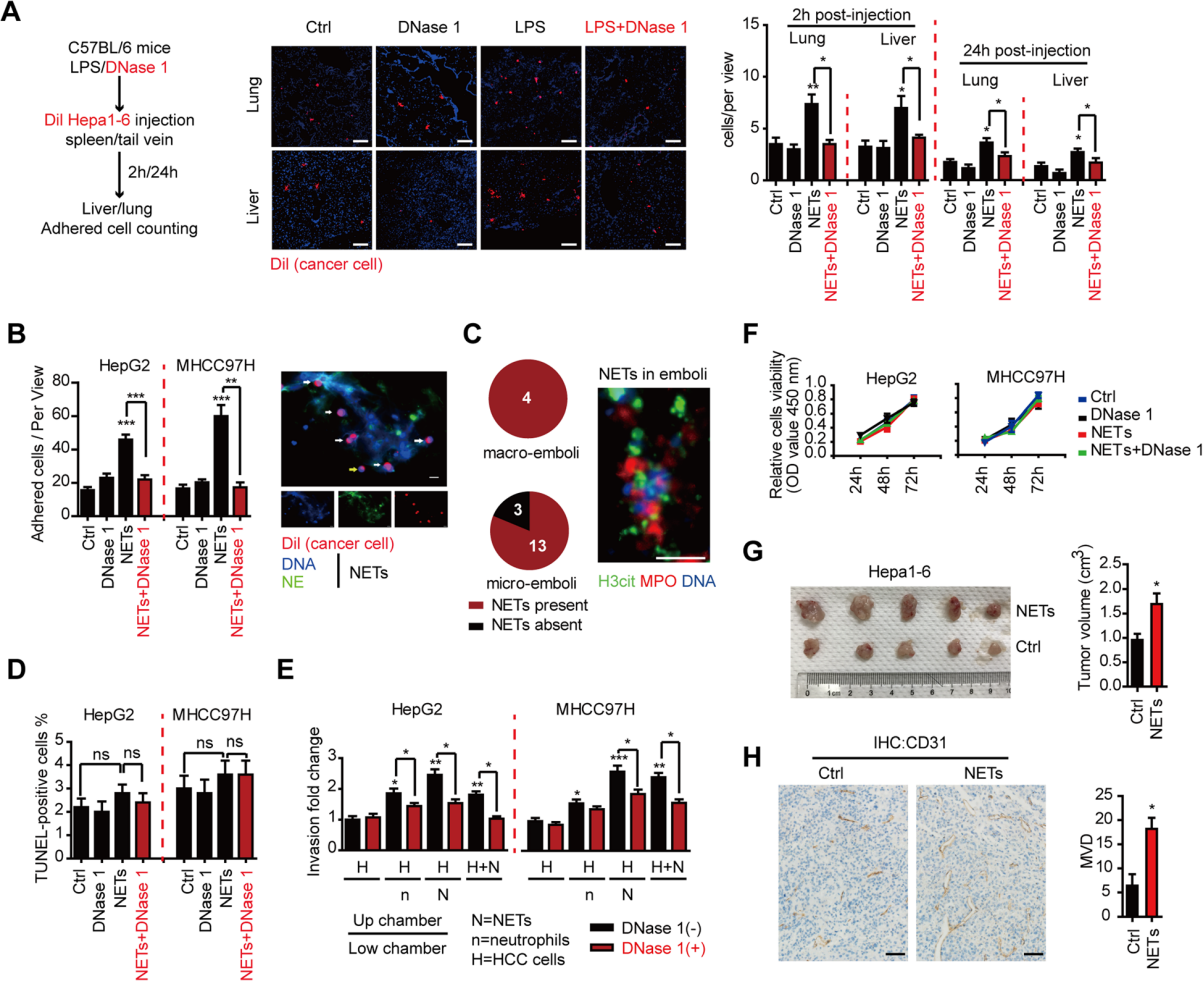
NETs optimized adhesion of HCC and further promoted metastasis potential by raising invasiveness with minimal cytotoxicity. **a** Representative fluorescence images (middle panel, 2 h post-injection) and quantification (right panel) of early trapped HCC cells in the lung and liver in LPS-induced NET model. C57BL/6 mice were subjected to systemic LPS and/or DNase 1 abrogation, and subsequently intraspleen/intravenous injected with Dil-labeled Hepa1-6 cells (*n* = 5 each). Scare bar: 50 μm. **b** Increased adhesion of Dil-labeled HepG2/MHCC97H cells within PMA-induced NETs in vitro, but not with intact neutrophils or NETs plus DNase 1 abrogation (left panel). Representative fluorescence image of HCC cells trapped within NETs in vitro was shown (right panel). Scare bar: 20 μm. **c** NET detection in HCC embolus and representative fluorescence image. Scare bar: 50 μm. **d** Little cytotoxicity on HepG2/MHCC97H cells with NET treatment or DNase 1 abrogation revealed by TUNEL assay. **e** Enhanced invasiveness of HepG2/MHCC97H cells with NETs under different conditions in a Transwell system as indicated, which was abrogated by DNase 1. Quantification of invading cells through Matrigel-coated PET membrane was shown. **f** Little alteration of in vitro proliferation capacity of HepG2/MHCC97H cells with NET treatment or DNase 1 abrogation in CCK8 assay. **g** Hepa1-6 subcutaneous tumor growth increased in the presence of NET-producing neutrophils from LPS-treated C57BL/6 mice in vivo (*n* = 5 each group). **h** Increased angiogenesis in NET-Hepa1-6 subcutaneous tumors compared to hepa1-6 alone. Representative images of CD31 staining were shown. Scare bar: 50 μm. *P < 0.05; **P < 0.01; ***P < 0.001; ns, no significance. Data were presented as means ± SEM

We next focused on the mechanism how NETs facilitated metastasis. We observed a significant increased adhesion of HCC cells trapped in liver and lung of the LPS-induced NETs model, and DNase 1 significantly diminished this increase (Fig. 3a). These were validated in a null-mice model co-implanted with induced human NETs and HepG2 cells in vitro (Additional file 2: Figure S8A). In line, HCC cells were tightly trapped by extensive NET structure in vitro (Fig. 3b, right panel). And NETs abrogated by DNase 1 or intact neutrophils without NET formation displayed little enhancing effect on cell adhesion (Fig. 3b and Additional file 2: Figure S8B). Moreover, NETs were also present in a large portion of vascular-thrombi of HCC tissues, which further supported the trapping role of NETs on HCC metastasis (Fig. 3c).

NETs may exert potential cytotoxicity on trapped HCC cells, as a similar pattern to that on entrapped pathogen, and serve as a “trap and kill” role to restrict metastasis by eliminating circulating HCC cells rather than promoting metastasis [23, 24]. However, no significant cytotoxicity of NETs was observed on HCC cells (Fig. 3d and Additional file 2: Figure S8C-D), and HCC cells gained resistance to the potential cytotoxicity once in contact with NETs. Furthermore, NETs efficiently promoted invasion of trapped HCC cells in the Transwell conditions, while DNase 1 diminished the enhanced invasion of HCC cells (Fig. 3e and Additional file 2: Figure S8E). We observed no significant alteration on proliferation of HCC cells after stimulation with NETs in vitro (Fig. 3f and Additional file 2: Figure S8F). But NETs could promote in vivo tumor growth, as the tumor volume was much larger when hepa1-6 HCC cells were subcutaneously implanted together with NETs into C57BL/6 mice than that of HCC cell implantation alone (Fig. 3g). Immunohistochemical staining demonstrated that the CD31 expression was much stronger in the tumors of NET-HCC co-implantation, which indicated that NETs accelerated HCC growth by promoting angiogenesis rather than direct proliferation capacity of HCC cells (Fig. 3h).

These findings suggested in addition to trapping, NETs could trigger resistance to potential cytotoxicity, enhance invasiveness and angiogenesis of the trapped HCC cells, thus facilitating HCC metastasis.

### NETs induce an inflammatory response in HCC cells featured as COX2 upregulation

**Fig. 4:**
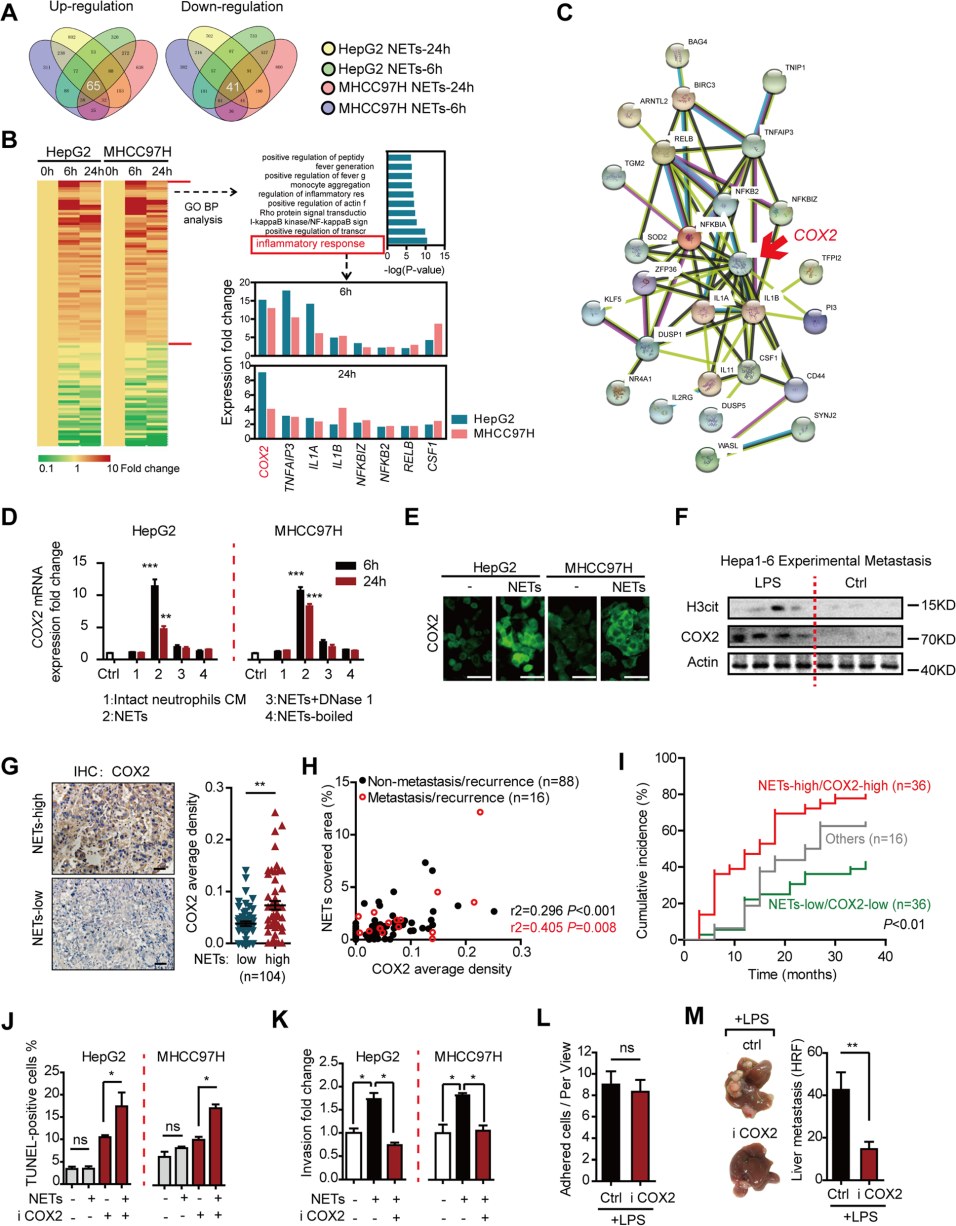
NETs triggered an enhanced metastasis potential by raising an inflammatory response headed by COX2 elevation. **a** Venn diagram of the common up/downregulated genes detected by RNA-seq in HepG2/MHCC97H cells treated with NETs. **b** Heatmap of altered genes and the GO biological process analysis. COX2 and other pro-inflammatory genes were robustly upregulated in response to NETs. **c** MeV software of protein network predicting COX2 at the central of NET-induced inflammatory response (http://string.embl.de/). **d** RT-PCR verification of upregulation of COX2 expression in HepG2 and MHCC97H in response to NETs, but not intact neutrophils or NETs with DNase 1 and boil abrogation. **e** Immunofluorescence assay verifying the upregulation of COX2 expression in HepG2 and MHCC97H cells. **f** Western blot showing upregulation of COX2 correlated with NET marker H3cit in metastasis liver lesions from LPS-induced NETs mice. **g** Representative images of higher COX2 expression in NET-high HCC tissues (identified by median of H3cit staining in Fig. 1h, *n* = 104) and statistic graph. Scare bar: 50 μm. **h** Pearson correlation analysis of NETs and COX2 expression in HCC samples (*n* = 104). **i** Follow-up data of the 88 non-metastasis/recurrence HCC patients with different NET and COX2 expression for metastasis/recurrence cumulative incidence analysis. Log-rank test was used. **j** An extruded cytotoxicity of NETs on cell death rate of HepG2/MHCC97H cells pretreated with COX2 inhibitor Celecoxib in TUNEL assay. **k** An inhibitory effect of Celecoxib on invasion capacity of HepG2/MHCC97H cells treated by NETs. **l** No change in early adhesion number of Celecoxib-treated hepa1-6 cells in LPS-induced NET mice. Dil-labeled Hepa1-6 cells were pretreated with Celecoxib followed by intraspleen injection, and liver frozen sections 2 h post-injection were observed under fluorescence microscope for early adhesion quantification (*n* = 5 each). **m** Less metastasis lesions formed by Celecoxib-treated hepa1-6 cells in LPS-induced NET mice model (*n* = 5 each). Saline served as control. *P < 0.05; **P < 0.01; ***P < 0.001. Data were presented as means ± SEM

To further elucidate the underlying mechanism of NETs facilitating HCC metastasis, we performed RNA-seq on HepG2 and MHCC97H cells under NET stimulation. Total 65 and 41 genes were consistently upregulated or downregulated in HCC cells with NETs, respectively (Fig. 4a). Gene Ontology Biological Process (GOBP) analysis suggested NETs triggered an inflammatory response in trapped HCC cells. A set of genes coding inflammatory mediators including IL-1α/β, CSF-1, and COX2 were consecutively upregulated (Fig. 4b), among which COX2 (cyclooxygenase-2, or PTGS2) displayed a marked and persistent trend and located at the center of predicted inflammatory response (Fig. 4c). RT-PCR was performed to validate the RNA-seq data (Additional file 2: Figure S9) and demonstrated that NET-induced COX2 upregulation was abrogated when NETs were degenerated by DNase 1 or totally wrecked by boil, which supported the upregulation of COX2 resulted from NETs rather than intact neutrophils (Fig. 4d). This was further validated at the post-transcriptional level in vitro (Fig. 4e) and in experimental metastases from LPS-induced NET mice (Fig. 4f). To be noticed, DNase 1 hardly altered any tumorous inflammatory genes level when it was applied alone (Additional file 2: Figure S9). In human HCC specimens, a close correlation between NET (marked as H3cit) and COX2 expression was also revealed (Fig. 4g, h). Moreover, patients with higher NET and COX2 expression had higher probabilities of metastatic/recurrence (Fig. 4i). In addition, the close correlation between infiltrated neutrophils/NETs and COX2 or other inflammatory genes was also found in TCGA and TIMER database, which further supported our observation (Additional file 2: Figure S10-11). Taken together, these findings indicate that NETs induce the upregulation of tumorous COX2.

We next illustrated the necessity of COX2 in NET-triggered metastatic capacity of the trapped HCC cells. As described above, HCC cells viability was not affected by NETs. However, HCC cells pretreated with Celecoxib (COX2 inhibitor) presented higher cell death rate after NET treatment, which extruded the potential cytotoxic effect of NETs on HCC cells in the presence of COX2 inhibitor (Fig. 4j). Celecoxib could also abrogate the enhanced invasion capacity of HCC cells stimulated with NETs (Fig. 4k). But, inhibitors of TNFα or IL-1 did not demonstrate these effects (Additional file 2: Figure S12A-B). In experimental metastasis assay using LPS-induced NET model, although HCC cells after Celecoxib pretreatment displayed no defect in early adhesion trapping by NETs (Fig. 4l), they formed remarkably less metastases (Fig. 4m). All together, these suggested that NETs facilitated metastasis by inducing an aggressive inflammatory response featured as COX2 upregulation in the trapped HCC cells.

### NETs enhance metastatic potential of the trapped HCC cells through activating TLR4/9

**Fig. 5:**
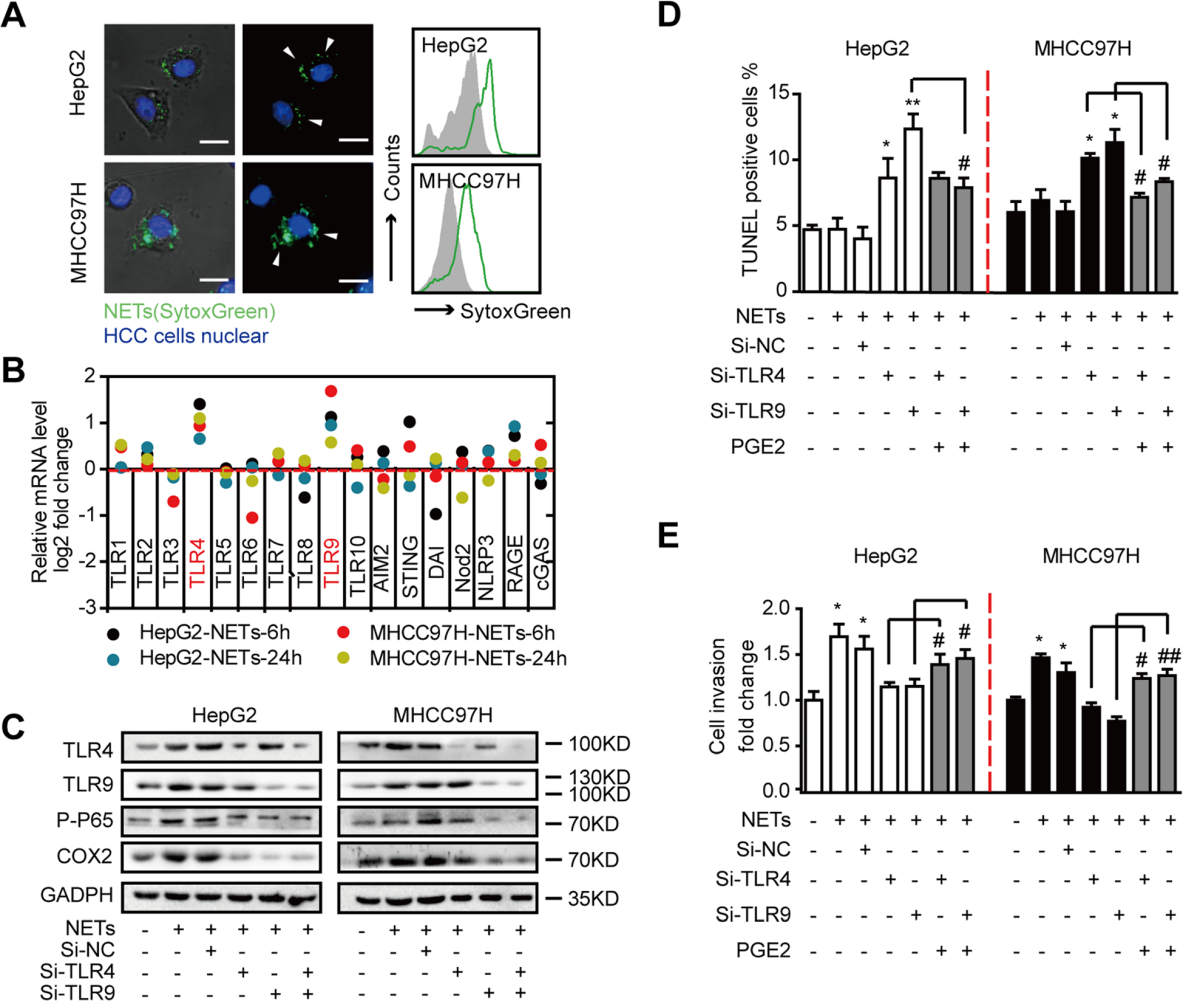
NETs internalized by HCC cells upregulated COX2 through activation of TLR4/9. **a** Representative fluorescent images of NET internalization by HepG2 and MHCC97H cells (left panel) and flow cytometry analysis (right panel). NETs were labeled with SytoxGreen and incubated with HepG2 and MHCC97H cells for 6 h. The HCC cells were then fixed, stained for nuclear, and observed under fluorescence microscope or analyzed by flow cytometry. The white triangle indicated NETs internalized. Scar bar: 20 μm. **b** RT-PCR screening of mRNA level changes in chosen DAMP sensors in HepG2 and MHCC97H cells treated with NETs. Results were presented as log_2_ fold change relative to untreated cells. **c** Western blot analysis of COX2, TLR4/9, and P-P65 levels in HepG2 and MHCC97H cells treated with NETs. Cells were transfected with corresponding siRNA and stimulated with NETs for 6 h. **d**, **e** The cytotoxicity (**d**) and invasion capacity alteration (**e**) on siRNA transferred HepG2/MHCC97H cells followed by NET treatment and PGE2 rescue. *, #: *P* < 0.05; **,##: *P* < 0.01. Data were presented as means ± SEM

To explore the mechanism of the NET-induced upregulation of COX2, we performed immunofluorescence staining and flow cytometry in the NET-trapped HCC cells and observed that the trapped HCC cells could conversely internalize part of NETs in vitro (Fig. 5a). We then detected the expression levels of several classical DAMP sensors that might play some roles in NET internalization. Among which, Toll-like receptor TLR4/9 mRNA level displayed a consistent trend of elevation in response to NET stimulation (Fig. 5b). This indicated their possible roles in NET internalization.

We then used siRNA to block TLR4/9 activity in HCC cells and found that NETs were insufficient to induce phosphorylation of NF-κB pathway (P65) and COX2 expression in HepG2^si-TLR4/9^ and MHCC97H^si-TLR4/9^ cells (Fig. 5c). NETs were not able to influence the resistance to NET potential toxic effects, and the invasion capacity of HCC cells transfected with si-TLR4/9. Treatment with prostaglandin E2 (PGE2), a direct product of COX2, could rescue the effects of NETs on HCC cells (Fig. 5d, e). In addition, hydroxychloroquine (HCQ), a well-applied anti-inflammation drug with TLR-pathway blockage capacity [25, 26], could effectively abrogate COX2 upregulation and the subsequent enhanced the metastatic behaviors of HCC cells induced by NETs (Additional file 2: Figure S13). These findings indicated that NETs stimulated the metastatic potential of the trapped HCC cells through activating TLR4/9 activity.

### Targeting NETs effectively inhibits HCC metastasis

**Fig. 6:**
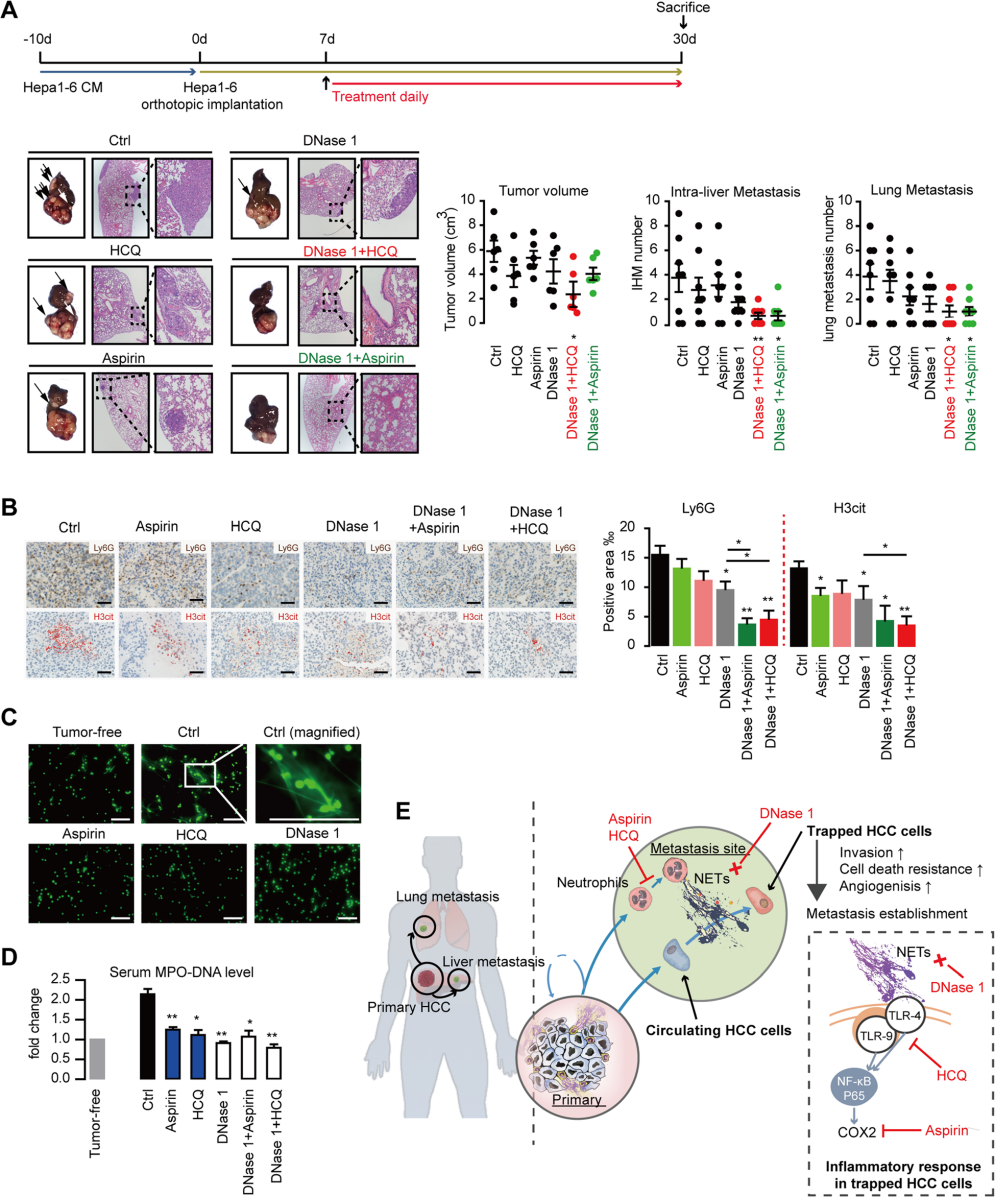
A combined strategy of DNase 1 plus HCQ/Aspirin against NETs effectively impaired HCC metastasis in a mice model. **a** Effect of DNase 1 and HCQ/Aspirin alone or in combination on HCC progression and spontaneous metastasis (*n* = 8 each) in a modified Hepa1-6 orthotopic model. Representative images of liver tumor with intrahepatic metastasis (IHM, pointed with a rightward arrow) and lung metastasis, and quantification were presented. **b** Representative images of H3cit and Ly6G expression and quantification. The expression of H3cit was stressed in red color by Adobe Photoshop software with identical procedure. Scare bar: 50 μm. **c** Representative fluorescence images of impaired NET formation from HCC-associated neutrophils treated with HCQ or Aspirin in vitro. Neutrophils were isolated from peripheral blood of Hepa1-6 orthotopically implanted mice, incubated for 4 h for NET formation in the presence of HCQ, Aspirin, or DNase 1, fixed and stained for DNA with SytoxGreen. Scare bar: 100 μm. **d** Impaired NET formation by Aspirin or HCQ plus DNase 1 treatment in vivo by serum MPO-DNA level. **e** System illustration of NETs to trap and fuel HCC metastasis potential through triggering an inflammatory response through TLR4/9-COX2 signaling. *P < 0.05; **P < 0.01. Data were presented as means ± SEM

The role of NETs on HCC progression and metastasis implicates its potential therapeutic potential. DNase 1, which has been applied clinically in autoimmune disease with little toxicity, could degenerate the massive formation of NETs induced by systemic LPS addition, and effectively abolished the experimental metastasis of HCC (Fig. 2e, f). However, under physiology condition, the release of cancer cells into circulation is a continual process rather than loading of large number of tumor cells at once. Thus, a subsequent intervention against NET-induced inflammatory response in HCC cells might be of reinforcement compared with DNase 1 treatment alone against HCC metastasis. Aspirin and HCQ, two clinical well-applied anti-inflammation medications with acceptable side-effects, can inhibit NET-triggered metastatic capacity by targeting COX2 or blocking upstream TLR. We then adopted DNase 1 in combination with HCQ or Aspirin against NETs in the orthotopic mice model of HCC and found this combination displayed a more significant efficiency in inhibiting the spontaneous intra-hepatic and lung metastasis of HCC compared with DNase 1 alone (Fig. 6a). We discovered aspirin and HCQ displayed some abrogation of the NET formation both in vivo and in isolated neutrophils (Fig. 6b, c, d), which was consistent with reports in other diseases. In line with the in vitro results, the combined therapy targeting NETs proved effective in decreasing several tumorous inflammatory mediators (Additional file 2: Figure S14). Taken together, DNase 1 in combination with HCQ or Aspirin is more effective in inhibiting NET formation and could be a novel and practical strategy against HCC metastasis with acceptable side-effects (Fig. 6e).

## Discussion

Metastasis is a complex multistep cascade, which is related to both biological features of tumor cells and non-malignant tumor stroma. Accumulating evidences suggest that inflammation status of tumor microenvironment bridges host and cancer cells to affect metastasis cascade [1]. Neutrophils, the most abundant host inflammatory cells, may influence multiple steps of metastasis. Neutrophils are often found in high numbers in human tumors and mice models, but there is a controversy with their roles in metastasis, depending on the different neutrophil subtypes or the different tumor types and the microenvironment studied [7]. Neutrophils are found to be accumulated in HCC and associated with a worse outcome [18]. Some non-specific inflammatory mediators from neutrophils have been found to influence HCC progression [19, 27]. However, the specific functions of neutrophils in HCC metastasis remains to be illustrated. In this study, we demonstrated that neutrophils promoted HCC metastasis through forming enhanced NETs, which trapped HCC cells and further provoked their metastasis potential. Mechanically, NETs triggered a tumorous inflammatory response through activation of TLR4/9-COX2 axis to fuel metastasis.

NET formation is a unique functional process of neutrophils first described in host defense to trap and kill invading pathogen, with emerging recognition of which in non-infectious diseases and sterile inflammation [10, 11]. The important roles of NETs have been described in some kinds of solid malignancies [13, 15, 28]. One study has linked sterile inflammation-driven NETs with HCC tumorigenesis in mice with steatohepatitis [29]. But the roles of NETs in HCC metastasis remain to be illustrated. In the present study, through various detection means including isolated neutrophils, sera and pathological samples in both mice models and human patients, we provide solid evidence to support that NET formation of neutrophils is enhanced in patients with HCC, especially those with metastatic HCCs. However, the really involved mechanism is not understood. We proposed that several secreted factors from HCC cells activated neutrophils towards NET formation or prime neutrophils for enhanced NETs with “second-hit” such as infection or stress. Some studies have indicated that NET-promoting effect is attributed to certain tumor-released cytokines or vesicles [30, 31]. However, the pattern of secreted factors is highly distinct among various cancers. When expanded beyond cancer, the range of NET-promoting factors may even cover chromatin and lipid products [32, 33]*.*

The link between NETs and metastasis is getting appreciated. Biologically, metastasis is a low-efficient process where most of the disseminated cancer cells fail to seed and cease following cascade. Accumulating studies have suggested a seeding-supporting role of NETs to optimize the early adhesion of tumor cells to favor metastasis in different mice models [14, 34*–*36]*. In consistent with these reports*, here we also proved the dominant role of NETs to trap more disseminated HCC cells from circulation was necessary for the establishment of experimental metastasis of HCC and further found this effect was diminished in the case with normal neutrophils or NETs disturbed. But how NETs facilitate metastasis after trapping tumor cells is largely less known. NETs are equipped with toxic protease that cause cell damage [24], which raises a possibility that NETs may restrict metastasis by killing trapped cancer cells with cytotoxicity in a similar pattern to eliminate pathogen [24, 28]. This possibility is excluded, since the present study has demonstrated that the trapped HCC cells are not affected by the potential cytotoxicity of NETs. And more, the invasiveness of HCC cells is enhanced after surviving from NETs. These suggest that certain key defense/survival event is triggered by NET challenge in trapped HCC cells which acquired a higher metastasis potential. The acquired invasiveness enhancement as a survival mechanism has been described in cancer cells upon potential deadly extracellular stress [37]. NETs may act as a beneficial stress on HCC cells in a similar pattern.

Then comes an interesting question that how these trapped HCC cells withstand and utilize NETs to enhance their metastatic potential. Here, we have found NETs induce an aggressive inflammatory response in the trapped HCC cells featured as COX2 upregulation through activating TLR4/9 to enhance metastatic potential of the trapped HCC cells. Through RNA-seq, we identified COX2 as the key event of NET-triggered metastasis potential. NETs are of strong immunostimulatory capacity and known to license macrophages and other host cells for cytokine production in vitro [38]. This pro-inflammatory effect of NETs is also reported in mice [39]. For the first time, we found the effects of NETs on inflammatory response in boosting metastasis behavior of cancer cells. Elevation of COX2 is associated with a higher metastasis behavior, including protection from cell death, induction of invasion, stimulation of angiogenesis, and inhibition of immunosurveillance [40–42]. COX2 is also an appealing therapeutic target, and targeting COX2 has certain anti-metastasis effects even as a single intervention alone [3]. We have found when COX2 is blocked, trapped HCC cells lose the counteraction to NET potential cytotoxicity and acquire no enhanced invasiveness from NETs. These strongly suggested the induction of COX2 as the key molecular event responsible for NET-enhanced metastatic capacity in trapped HCC cells. Moreover, we revealed activation of TLR4/9 as the intermediate link between NETs and induction of the inflammatory response in trapped HCC cells. TLR4/9 are important sensors of several damage-associated molecular patterns (DAMPs) and mediate cellular communication among host cells, and their activation also represents a highly metastatic phenotype [25, 43]. NETs contain several DAMPs that may be recognized by TLR4/9 [44]. TLR signals mediate the pro-inflammatory effect of NETs on several host cells [45, 46]. Many reports have demonstrated that NETs could upregulate TLR9 expression in colon cancer cells and that TLR is an upstream regulator of COX2 expression [36, 47, 48]. By blocking TLR4/9, we have found NETs failed to induce COX2 or trigger metastasis capacity in trapped HCC cells. These findings suggest TLR4/9 activation and subsequent COX2 induction as the key signaling in NET-triggered metastasis potential.

DNase 1 is well acknowledged to digest extracellular chromatin and NETs. Endogenous DNase 1 is a vital physiological regulation of NETs in host and in adequate clearance of NETs due to low level or bio-activity of endogenous DNase 1 which may lead to dysregulation of NETs, thus causing autoimmune disease and other inflammatory disorders [49*–*51]. Many studies have revealed an association of DNase 1 polymorphism with the susceptibility of autoimmune disease such as systemic lupus erythematosus (SLE), but the correlation between endogenous DNase 1 and cancer remains to be studied [52]. As a therapeutic mean, DNase 1 has demonstrated a satisfied effect in digesting NETs in several preclinical models and confirmed safety in cystic fibrosis and SLE [53]. However, DNase 1 alone against NETs has certain limitation. The blood concentration of given DNase 1 is found less stable [54]. Besides, the fact that DNase 1 dismantles NET structure but does not totally degrades protein components of NETs indicate its less effectiveness in abrogating NET-triggered inflammatory response [53]. Combination of DNase 1 and other available means provides a solution. Targeting COX2 has been well acknowledged to have both anti-inflammatory capacity and anti-tumor effect through multiple mechanisms [3]. A capacity of anti-inflammatory drugs to decrease NETs is also demonstrated [40]. Based on our finding of NETs fueling HCC metastasis through activating tumorous inflammatory response, we adopted anti-inflammatory drugs aspirin and HCQ to block COX2 and upstream TLR4/9 activation complementary to DNase 1 and proved well efficiency in inhibition of HCC metastasis through multiple perspectives. These combination therapies could block or digest NETs and abrogate the triggered metastasis potential of trapped HCC cells by undissolved NETs, featuring a new use of old anti-inflammatory drugs. More combination strategies with DNase 1 against metastasis are to be developed.

## Conclusions

Our study demonstrated NET formation was increased in neutrophils derived from patients with HCC, especially those with metastatic HCC. The increased NETs not only trapped HCC cells but further induced cell-death resistance and enhanced invasion capacity to trigger their metastatic potential, which was marked as a provoked inflammatory response via internalization of NETs into HCC cells and activation of Toll-like receptors TLR4/9-COX2 signaling. Notably, abolishing the provoked inflammatory response by blocking TLR4/9-COX2 signaling abrogated the NET-aroused metastatic potential. A combination of DNase 1 directly wrecking NETs with anti-inflammation drugs aspirin/HCQ effectively reduced HCC metastasis in mice model. Our study highlights the role of NETs in HCC metastasis, which can serve as a novel therapeutic strategy against metastasis.

## Supplementary information

**Additional file 1.** Supplementary methods.

**Additional file 2: Figure S1.** NETs formation was enhanced in HCC associated neutrophils. **Figure S2.** Depositions of NETs was correlated with metastasis burden in HCC. **Figure S3.** NETs in HCC bearing mice. **Figure S4.** Evidence of NETs formation in TCGA and The Human Protein Atlas database. **Figure S5.** HCC enhanced the NETs formation capacity of normal neutrophils. **Figure S6.** NETs formation was enhanced in HCC-driven inflammatory condition in mice. **Figure S7.** Effect of DNase 1 and neutrophil depletion on LPS-induced NETs in mice. **Figure S8.** NETs trap optimized adhesion of HCC, and further promoted metastasis potential by raising invasion capacity with minimal cytotoxicity. **Figure S9.** Effect of DNase 1 on inflammatory mediators expression in NETs-stimulated HCC cells. **Figure S10.** Correlation analysis of infiltrated neutrophils and COX2 or other inflammatory mediator genes in TIMER database. **Figure S11.** Pearson correlation analysis of PADI4/MPO and COX2 or other inflammatory mediator genes in TCGA database. **Figure S12.** Effects of inhibiting IL-1 and TNF-α on NETs-aroused metastasis potential. **Figure S13.** HCQ impaired the enhanced metastasis potential triggered by NETs. **Figure S14.** Targeting NETs abrogated tumorous inflammatory response.

**Additional file 3: Table S1.** Clinicopathological characteristics of HCC patients for NETs pathological analysis (n = 104). **Table S2.** Clinicopathological characteristics of HCC patients for serum MPO-DNA detection (n = 73). **Table S3.** Real-time PCR primers used in the study. **Table S4.** Primary antibodies used in the study.

## Data Availability

All data generated or analyzed during this study are included in this published article and its additional files.

## Abbreviations

CM: Conditioned medium
COX2: Cyclooxygenase 2
HCC: Hepatocellular carcinoma
HCQ: Hydroxychloroquine
HD: Healthy donor
LPS: Lipopolysaccharide
MPO: Myeloperoxidase
NE: Neutrophil elastase
NETs: Neutrophil extracellular traps
PGE2: Prostaglandin E2
PMA: Phorbol 12-myristate 13-acetate
TLR: Toll-like receptor

## References

[1] Lambert AW, Pattabiraman DR, Weinberg RA (2017). Emerging biological principles of metastasis. Cell.

[2] Quail DF, Joyce JA (2013). Microenvironmental regulation of tumor progression and metastasis. Nat Med.

[3] Dannenberg AJ, Subbaramaiah K (2003). Targeting cyclooxygenase-2 in human neoplasia: rationale and promise. Cancer Cell.

[4] Tuting T, de Visser KE (2016). CANCER. How neutrophils promote metastasis. Science.

[5] Coffelt SB, Wellenstein MD, de Visser KE (2016). Neutrophils in cancer: neutral no more. Nat Rev Cancer.

[6] Mayadas TN, Cullere X, Lowell CA (2014). The multifaceted functions of neutrophils. Annual Review of Pathology: Mechanisms of Disease.

[7] Fridlender ZG, Albelda SM (2012). Tumor-associated neutrophils: friend or foe?. Carcinogenesis.

[8] Granot Z, Henke E, Comen EA (2011). Tumor entrained neutrophils inhibit seeding in the premetastatic lung. Cancer Cell.

[9] Wculek SK, Malanchi I (2015). Neutrophils support lung colonization of metastasis-initiating breast cancer cells. Nature.

[10] Brinkmann V, Reichard U, Goosmann C (2004). Neutrophil extracellular traps kill bacteria. Science.

[11] Jorch SK, Kubes P (2017). An emerging role for neutrophil extracellular traps in noninfectious disease. Nat Med.

[12] Papayannopoulos V (2018). Neutrophil extracellular traps in immunity and disease. Nat Rev Immunol.

[13] Berger-Achituv S, Brinkmann V, Abed UA, et al. A proposed role for neutrophil extracellular traps in cancer immunoediting. Front Immunol. 2013;4. 10.3389/fimmu.2013.00048 PMC3589747 23508552

[14] Cools-Lartigue J, Spicer J, McDonald B (2013). Neutrophil extracellular traps sequester circulating tumor cells and promote metastasis. J Clin Invest.

[15] Demers M, Krause DS, Schatzberg D (2012). Cancers predispose neutrophils to release extracellular DNA traps that contribute to cancer-associated thrombosis. Proc Natl Acad Sci U S A.

[16] Park J, Wysocki RW, Amoozgar Z (2016). Cancer cells induce metastasis-supporting neutrophil extracellular DNA traps. Sci Transl Med.

[17] Llovet JM, Montal R, Sia D, Finn RS (2018). Molecular therapies and precision medicine for hepatocellular carcinoma. Nature reviews. Clin Oncol.

[18] Zhou SL, Dai Z, Zhou ZJ (2012). Overexpression of CXCL5 mediates neutrophil infiltration and indicates poor prognosis for hepatocellular carcinoma. Hepatology.

[19] Li XF, Chen DP, Ouyang FZ (2015). Increased autophagy sustains the survival and pro-tumourigenic effects of neutrophils in human hepatocellular carcinoma. J Hepatol.

[20] Kuang DM, Zhao Q, Wu Y (2011). Peritumoral neutrophils link inflammatory response to disease progression by fostering angiogenesis in hepatocellular carcinoma. J Hepatol.

[21] Kessenbrock K, Krumbholz M, Schönermarck U (2009). Netting neutrophils in autoimmune small-vessel vasculitis. Nat Med.

[22] Tanaka K, Koike Y, Shimura T (2014). In vivo characterization of neutrophil extracellular traps in various organs of a murine sepsis model. PLoS One.

[23] Carmona-Rivera C, Zhao W, Yalavarthi S, Kaplan MJ (2015). Neutrophil extracellular traps induce endothelial dysfunction in systemic lupus erythematosus through the activation of matrix metalloproteinase-2. Ann Rheum Dis.

[24] Saffarzadeh M, Juenemann C, Queisser MA (2012). Neutrophil extracellular traps directly induce epithelial and endothelial cell death: a predominant role of histones. PLoS One.

[25] Thierry AR, El MS, Gahan PB, Anker P, Stroun M (2016). Origins, structures, and functions of circulating DNA in oncology. Cancer Metastasis Rev.

[26] Kuznik A, Bencina M, Svajger U (2011). Mechanism of endosomal TLR inhibition by antimalarial drugs and imidazoquinolines. J Immunol.

[27] Zhou S, Zhou Z, Hu Z (2016). Tumor-associated neutrophils recruit macrophages and T-regulatory cells to promote progression of hepatocellular carcinoma and resistance to Sorafenib. Gastroenterology.

[28] Arelaki S, Arampatzioglou A, Kambas K (2016). Gradient infiltration of neutrophil extracellular traps in colon cancer and evidence for their involvement in tumour growth. PLoS One.

[29] van der Windt DJ, Sud V, Zhang H (2018). Neutrophil extracellular traps promote inflammation and development of hepatocellular carcinoma in nonalcoholic steatohepatitis. Hepatology.

[30] Gomes T, Varady C, Lourenco AL (2019). IL-1beta blockade attenuates thrombosis in a neutrophil extracellular trap-dependent breast cancer model. Front Immunol.

[31] Leal AC, Mizurini DM, Gomes T (2017). Tumor-derived exosomes induce the formation of neutrophil extracellular traps: implications for the establishment of cancer-associated thrombosis. Sci Rep.

[32] Palladino E, Katunga LA, Kolar GR, Ford DA (2018). 2-Chlorofatty acids: lipid mediators of neutrophil extracellular trap formation. J Lipid Res.

[33] Itagaki K, Kaczmarek E, Lee YT (2015). Mitochondrial DNA released by trauma induces neutrophil extracellular traps. PLoS One.

[34] Lee W, Ko SY, Mohamed MS (2019). Neutrophils facilitate ovarian cancer premetastatic niche formation in the omentum. J Exp Med.

[35] Inoue M, Nakashima R, Enomoto M (2018). Plasma redox imbalance caused by albumin oxidation promotes lung-predominant NETosis and pulmonary cancer metastasis. Nat Commun.

[36] Tohme S, Yazdani HO, Al-Khafaji AB (2016). Neutrophil extracellular traps promote the development and progression of liver metastases after surgical stress. Cancer Res.

[37] Tuomela J, Sandholm J, Kaakinen M (2013). DNA from dead cancer cells induces TLR9-mediated invasion and inflammation in living cancer cells. Breast Cancer Res Tr.

[38] Warnatsch A, Ioannou M, Wang Q, Papayannopoulos V (2015). Neutrophil extracellular traps license macrophages for cytokine production in atherosclerosis. Science.

[39] Luo L, Zhang S, Wang Y (2014). Proinflammatory role of neutrophil extracellular traps in abdominal sepsis. AJP: Lung Cellular and Molecular Physiology.

[40] Kern MA, Haugg AM, Koch AF (2006). Cyclooxygenase-2 inhibition induces apoptosis signaling via death receptors and mitochondria in hepatocellular carcinoma. Cancer Res.

[41] Leng J (2003). Cyclooxygenase-2 promotes hepatocellular carcinoma cell growth through Akt activation: evidence for Akt inhibition in celecoxib-induced apoptosis. Hepatology.

[42] Xu L, Stevens J, Hilton MB (2014). COX-2 inhibition potentiates antiangiogenic cancer therapy and prevents metastasis in preclinical models. Sci Transl Med.

[43] Dajon M, Iribarren K, Cremer I (2017). Toll-like receptor stimulation in cancer: a pro- and anti-tumor double-edged sword. Immunobiology.

[44] Urban CF, Ermert D, Schmid M (2009). Neutrophil extracellular traps contain calprotectin, a cytosolic protein complex involved in host defense against Candida albicans. PLoS Pathog.

[45] An Z, Li J, Yu J (2019). Neutrophil extracellular traps induced by IL-8 aggravate atherosclerosis via activation NF-kappaB signaling in macrophages. Cell Cycle.

[46] Gestermann N, Di Domizio J, Lande R (2018). Netting neutrophils activate autoreactive B cells in lupus. J Immunol.

[47] Lin A, Wang G, Zhao H (2015). TLR4 signaling promotes a COX-2/PGE2/STAT3 positive feedback loop in hepatocellular carcinoma (HCC) cells. Oncoimmunology.

[48] Yeo SJ, Gravis D, Yoon JG, Yi AK (2003). Myeloid differentiation factor 88-dependent transcriptional regulation of cyclooxygenase-2 expression by CpG DNA: role of NF-kappaB and p38. J Biol Chem.

[49] Tinazzi E, Puccetti A, Gerli R (2009). Serum DNase I, soluble Fas/FasL levels and cell surface Fas expression in patients with SLE: a possible explanation for the lack of efficacy of hrDNase I treatment. Int Immunol.

[50] Nakazawa D, Shida H, Tomaru U (2014). Enhanced formation and disordered regulation of NETs in myeloperoxidase-ANCA-associated microscopic polyangiitis. J Am Soc Nephrol.

[51] Hakkim A, Furnrohr BG, Amann K (2010). Impairment of neutrophil extracellular trap degradation is associated with lupus nephritis. Proc Natl Acad Sci U S A.

[52] Fujihara J, Ueki M, Kimura-Kataoka K (2016). Functional single nucleotide polymorphisms (SNPs) in the genes encoding the human deoxyribonuclease (DNase) family potentially relevant to autoimmunity. Immunol Investig.

[53] Honda M, Kubes P (2018). Neutrophils and neutrophil extracellular traps in the liver and gastrointestinal system. Nat Rev Gastroenterol Hepatol.

[54] Prince WS, Baker DL, Dodge AH (1998). Pharmacodynamics of recombinant human DNase I in serum. Clin Exp Immunol.

