# Supplementary material for: Increased neutrophil extracellular traps promote metastasis potential of hepatocellular carcinoma via provoking tumorous inflammatory response
Source: J Hematol Oncol. 2020 Jan 6;13:3. doi: 10.1186/s13045-019-0836-0 (PMC6945602; doi:10.1186/s13045-019-0836-0)
Supplement: Supplementary file 1 — Additional file 1. Supplementary methods. [file 13045_2019_836_MOESM1_ESM.doc]

**Supplementary Methods**

**Human specimens**

Paraffin-fixed HCC and paired non-tumor liver tissue were obtained from a total of 104 patients who underwent liver resection for primary onset or metastasis/recurrence HCC in our institute. Peripheral blood samples were collected from 73 HCC patients and 43 healthy donors (HD) for isolation of neutrophils or detection of serum MPO-DNA level. All samples were obtained under the regulation of the Ethics Committee of Huashan Hospital, Fudan University in agreement with the Declaration of Helsinki with written consent. Detail information of HCC patients is described in **Supplementary Table 1 and 2.**

**Animals, cell lines and condition medium (CM)**

Six- to 8-weeks old of C57BL/6 male mice or null-mice were obtained from Shanghai Slac Laboratory Animal Co. and fed under standard conditions. All animal experiments were approved by the Animal Ethics Committee of Fudan University. The human cell line L02, HepG2 and were obtained from Chinese Academy of Sciences. The human cell line MHCC97H and mice cell line Hepa1-6 were obtained from Liver Cancer Institute, Fudan University. All human cell lines were authenticated using STR profiling. HCC cell lines were cultured in DMEM supplemented with 10% FBS (Gibco), 1% penicillin/streptomycin at 37°C in a 5% CO_2_ incubator. **Dil (1:10000, Invitrogen)** was used for cell labeling following instructions. To establish TLR4/9 knockdown cells, siRNA specific for TLR4/9 (Santa Cruz Biotechnology) was transfected into cells with Lipofectin 2000. To generate condition medium (CM), HCC cells culture medium was discharged and replaced by serum-free DMEM overnight. Thereafter the supernatants were harvested, centrifuged and stored at -80°C for further use.

**Isolation of human and mice neutrophils**

Neutrophils were isolated from the blood obtained from HCC patients and healthy donors by a widely used one-step gradient centrifugation method using Polymorph ^Prep^ (Axis-Shield) according to instruction, and maintained in RPMI 1640 supplemented with 5% fetal calf serum (FCS) for immediate use. A purity of 90% was confirmed by flow cytometry using anti-CD15 antibody staining (BD Bioscience), with a viability rate over 95% by Trypan blue exclusion. Peripheral blood was obtained from mice through intracardiac extraction. Neutrophils were isolated through density gradient centrifugation using Histopaque 1077/1119 and maintained in PRMI as described above. A purity over 86% was confirmed by flow cytometry using anti-Ly6G antibody staining (BD Bioscience), and a viability rate over 95%.

***In vitro* assays on invasion, death rate, adhesion and proliferation of HCC cells**

In the set of *in vitro* invasion assay, 1×10^5^ HCC cells in serum-free DMEM were seeded on the upper chamber of 8-μm Transwell system coated with Matrigel (BD), and 5×10^5^ neutrophils/NETs with or without DNase 1 (100U/mL), Celecoxib (100 μM, R&D), IL-1R antagonist Anakinra (100ng/mL, Swedish Orphan Biovitrum AB), TNF-neutralizing agent Infliximab (10 μg/mL, Pfizer) and HCQ (dose gradient) were used as stimulus or inhibitors as indicated. After 30 hours incubation, the contents of the upper chambers were aspirated, washed and cleared by a cotton swab. Cells on lower membranes were then stained with crystal violet. Cells that invaded through the membrane were quantified in 4 random fields.

**The effect of NETs on HCC cell death was studied by Terminal-Deoxynucleotidyl Transferase Mediated Nick End Labeling (TUNEL) staining using the Cell Death Detection Kit (Roche) according to the manufacturer’s protocol. Briefly, 1×10^5^ HCC cells were seeded on 24-well plates and incubated overnight. NETs with or without DNase 1 (100U/mL) were added to each well and incubated with HCC cells for 24 hours. Celecoxib, Anakinra, Infliximab and HCQ were used 60 minutes prior to NETs treatment in selected experimental groups. The cells were fixed with 4% paraformaldehyde for 30 min and washed twice with PBS. Fixed cells were then washed and incubated with TUNEL assay mixture for 60 min. Then DAPI staining was followed by a final PBS wash, and TUNEL positive cells were examined under fluorescence microscopy. The average number of fluorescent dots in three images from each treatment group was calculated.**

For adhesion assay, 1×10^6^ human neutrophils were seeded on a 24-well plate and left intact or stimulated with PMA (20 nM) with or without DNase 1 (100U/mL) for 4 hours to form NETs, then 1×10^5^ Dil-labeled cells were added to each well. After incubation for 20 minutes, each well was washed five times, followed by 4% paraformaldehyde fixation. The adhered Dil-labeled cells were directly quantified under fluorescence microscopy from 5 random fields.

**The cell proliferation was studied using Cell Counting Kit-8 (CCK8) (Beyotime) according to instruction. Briefly, 2×10^3^ HCC cells were seeded on 96-well plates and incubated overnight. NETs with or without DNase 1 (100U/mL) were added to each well and incubated with HCC cells. The proliferation was measured every 24 hours and lasted for 72 hours. Two hours before the assay at each point, CCK-8 reagent (1:10) was incubated with the cells for 2 hours. The results were then measured using a microplate reader at 450 nm (optical density, OD). The relative proliferation activity was measured as treatment group OD/blank control group OD.**

**Cell immunofluorescence staining**

For NETs detection, 2×10^5^ neutrophils were seeded on poly-L-lysine–coated coverslips in 24-well plates to form NETs as described above, and then fixed with 4% paraformaldehyde for 20 minutes. Subsequently, coverslips containing NETs were permeabilized with 0.1% Triton X-100 for 15 minutes at RT, washed with phosphate-buffered saline, and blocked with PBS containing 1% bovine serum albumin for 1 hour at RT. NETs were stained with primary antibody in blocking buffer at 4°C overnight. After wash, NETs were stained with matched fluorescence-conjugated secondary antibodies (Jackson; 1:600) in blocking buffer and finally stained with Hoechst33342 for nuclear(1:1000). Slides were then mounted with Fluoro-gel (Beyotime) and observed under fluorescence microscopy. Images were analyzed with ImageJ software. A similar procedure was performed for immunofluorescence staining of HCC cells.

**Evaluation of *in vivo* adhesion of HCC cells in liver and lung**

**The evaluation of *in vivo* adhesion of cancer cells was referred to previous reports with some modifications[1-3].** Briefly, 5×10^5^ Dil-labeled Hepa1-6 cells were injected into the LPS-induced NETs models as described in establishment of LPS-induced NETs model. In a validation model using null-mice, 2×10^6^ PMA (20nM)-stimulated human neutrophils were given with or without DNase 1 pretreatment via intraspleen/intravenous injection in 20 minutes prior to 2×10^5^ Dil-labeled HepG2 cells. At indicated time point, lung and liver were removed, embedded in OCT and prepared for frozen sections. The average number of adhered cancer cells was counted in at least 10 random high field images from 5 sections under fluorescent microscope.

**Mice model: Subcutaneous implantation model**

To evaluate the effect of NETs on HCC growth, 1×10^6^ Hepa1-6 cells were subcutaneously implanted with the equal number of NETs-producing neutrophils from LPS-treated C57BL/6 mice. Tumor volume was measured in 20 days.

**Mice model: Modified spontaneous metastasis model**

1×10^6^ Hepa1-6 cells were subcutaneously implanted in C57BL/6 mice till tumor grew to 1cm in diameter. Meanwhile Hepa1-6 CM was intraperitoneally given into 6 weeks-old C57BL/6 mice for 10 days to generate an cancer-associated inflammation favoring NETs formation[4]. Subcutaneous tumor was then cut into pieces of 1mm and orthotopically implanted into the liver. Treatment of DNase 1 (100U/mouse), Aspirin (15 mg/kg) and HCQ (60mg/kg) alone or in combination started daily through peritoneal from day 7 after implantation till day 30. Saline served as control.

**Tissue immunohistochemical and immunofluorescence staining**

Immunohistochemical staining of paraffin-embedded sections was performed by the avidin-biotin-peroxidase complex method. Briefly, after rehydration and microwave antigen retrieval, primary antibodies were applied, incubated at 4°C overnight, and followed with secondary antibody incubation (GeneTech) at 37°C for 30 minutes. Staining was performed with 3, 30-diaminobenzidine tetra hydrochloride and counterstaining was performed with Mayer’s hematoxylin. Immunofluorescence staining of NETs components in paraffin-embedded sections was performed similarly. Sections were proceeded with rehydration and antigen retrieval, followed by elimination of auto-fluorescence. Primary antibodies were then applied, incubated at 4°C overnight, and followed with fluorescence-conjugated secondary antibody incubation and Hoechst33342 stain of nuclear.

**Evaluation of immunohistochemical and immunofluorescence variables**

Immunohistochemical and immunofluorescence results were assessed by 2 independent investigators. Photographs of 5 representative fields were captured and analyzed using software ImageJ with identical setting. NETs (marked as H3cit) and Ly6G were evaluated as percentage covered by positive staining area. NETs staining high/ low was identified by median. CD66b neutrophils and MVD (CD31) were calculated as numbers per field. COX2 staining was measured as average density.

**Quantitative real-time PCR and RNA-seq**

Total RNA was extracted using Trizol (Invitrogen), and reverse-transcribed into single-stranded cDNA using PrimeScript™ RT Reagent Kit (TaKaRa Biotechnology). Quantitative real time polymerase chain reaction (qRT-PCR) was performed with SYBR Green qPCR Master Mix (DBI Bioscience). Specific primers were provided in **Supplementary Table S3**. Expression levels were normalized against *β-actin* in each sample and then standardized as fold change.

**For RNA-seq assay, after incubation with NETs in different time points, total RNA of HepG2 and MHCC97H cells were isolated. rRNA was removed using RiboZero Magnetic Gold Kit (Illumina), and the purified mRNA was pooled to construct the cDNA library using KAPA_ Stranded RNA-Seq Library Prep Kit (Illumina) according to the manufacturer’s instructions. cDNA library was denatured by 0.1 M NaOH, and its amplification was conducted utilizing TruSeq SR Cluster Kit v3-cBot-HS (Illumina). Libraries were sequenced on a HiSeq 4000 (Illumina). A standard QC pipeline and reads mapping were performed on FastQC, followed by Hisat2. FPKM values were extracted and compared via Ballgown software. Total deferentially expressed RNA Seq reads for the transcripts were subjected to Gene Ontology analysis and hierarchical clustering analysis. Heatmap showing differential genes and their enriched pathways were plotted with R and related Bioconductor packages.**

**Immunoblotting**

Cells were lysed, and proteins were separated on 10%-15% SDS/PAGE gel and transferred onto PVDF membranes using a Mini-Trans Blot Electrophoretic Transfer System. The membranes were washed, blocked, and incubated with primary antibodies in 4℃ overnight, then the blots were washed, incubated with horseradish-peroxidase–conjugated secondary antibodies, and detected by ECL assays.

**Flow cytometry**

Single cell suspension was prepared from the lung and liver of mice, stained and analyzed by flow cytometry and flowJo software.

**References:**

1. Huh SJ, Liang S, Sharma A, Dong C, Robertson GP. Transiently Entrapped Circulating Tumor Cells Interact with Neutrophils to Facilitate Lung Metastasis Development. CANCER RES 2010;70:6071-6082.

2. Cools-Lartigue J, Spicer J, McDonald B, Gowing S, Chow S, Giannias B, Bourdeau F, Kubes P, Ferri L. Neutrophil extracellular traps sequester circulating tumor cells and promote metastasis. J CLIN INVEST 2013;123:3446-3458.

3. Labelle M, Begum S, Hynes RO. Platelets guide the formation of early metastatic niches. Proceedings of the National Academy of Sciences 2014;111:E3053-E3061.

4. Lee E, Pandey NB, Popel AS. Pre-treatment of mice with tumor-conditioned media accelerates metastasis to lymph nodes and lungs: a new spontaneous breast cancer metastasis model. Clin Exp Metastasis 2014;31:67-79.
