# Supplementary figures and images for: Increased neutrophil extracellular traps promote metastasis potential of hepatocellular carcinoma via provoking tumorous inflammatory response
Source: J Hematol Oncol. 2020 Jan 6;13:3. doi: 10.1186/s13045-019-0836-0 (PMC6945602; doi:10.1186/s13045-019-0836-0)

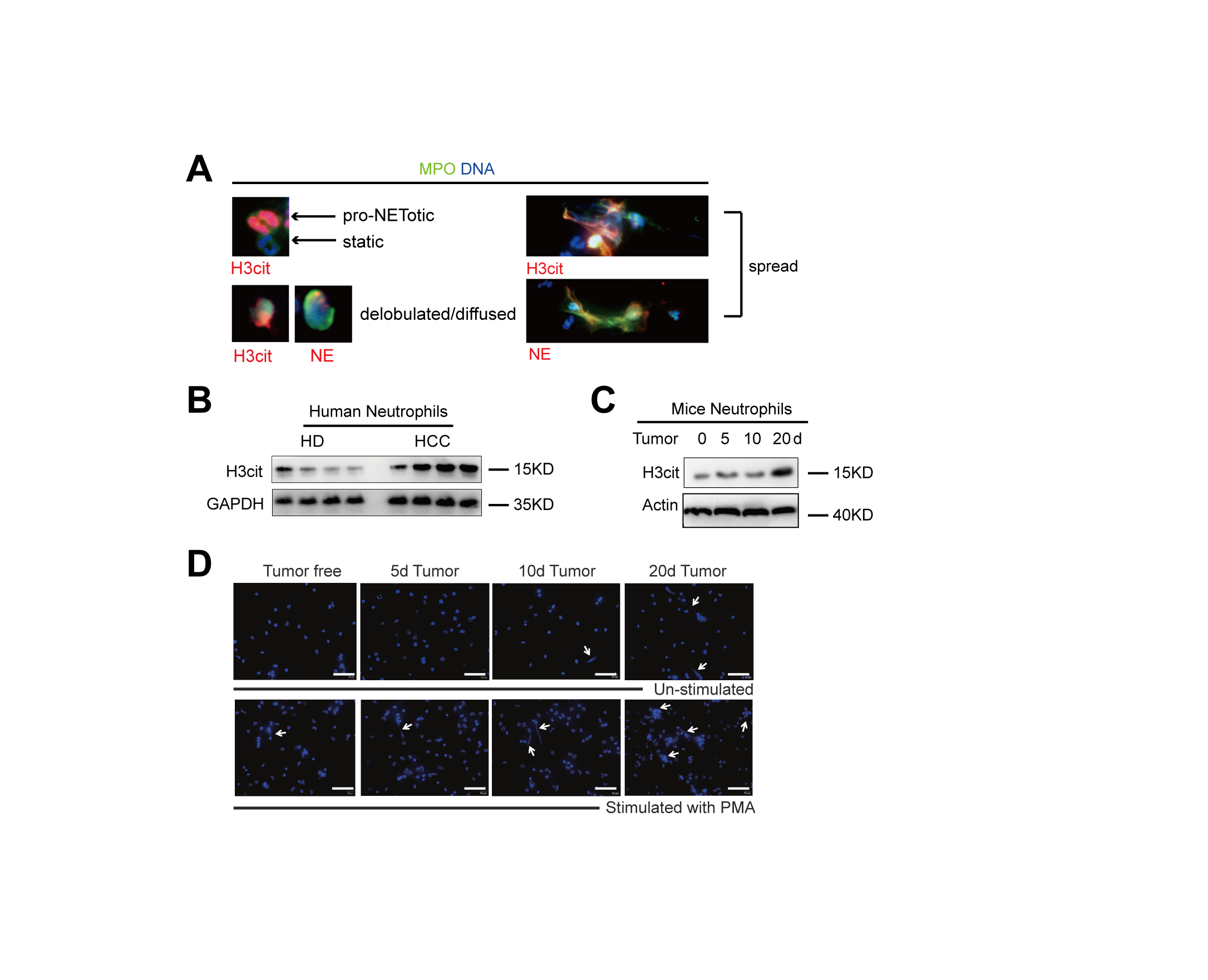

Supplement: Supplementary file 2 — Additional file 2: Figure S1. NETs formation was enhanced in HCC associated neutrophils. Figure S2. Depositions of NETs was correlated with metastasis burden in HCC. Figure S3. NETs in HCC bearing mice. Figure S4. Evidence of NETs formation in TCGA and The Human Protein Atlas database. Figure S5. HCC enhanced the NETs formation capacity of normal neutrophils. Figure S6. NETs formation was enhanced in HCC-driven inflammatory condition in mice. Figure S7. Effect of DNase 1 and neutrophil depletion on LPS-induced NETs in mice. Figure S8. NETs trap optimized adhesion of HCC, and further promoted metastasis potential by raising invasion capacity with minimal cytotoxicity. Figure S9. Effect of DNase 1 on inflammatory mediators expression in NETs-stimulated HCC cells. Figure S10. Correlation analysis of infiltrated neutrophils and COX2 or other inflammatory mediator genes in TIMER database. Figure S11. Pearson correlation analysis of PADI4/MPO and COX2 or other inflammatory mediator genes in TCGA database. Figure S12. Effects of inhibiting IL-1 and TNF-α on NETs-aroused metastasis potential. Figure S13. HCQ impaired the enhanced metastasis potential triggered by NETs. Figure S14. Targeting NETs abrogated tumorous inflammatory response. [file 13045_2019_836_MOESM2_ESM.zip › S-Figure 1.tif]

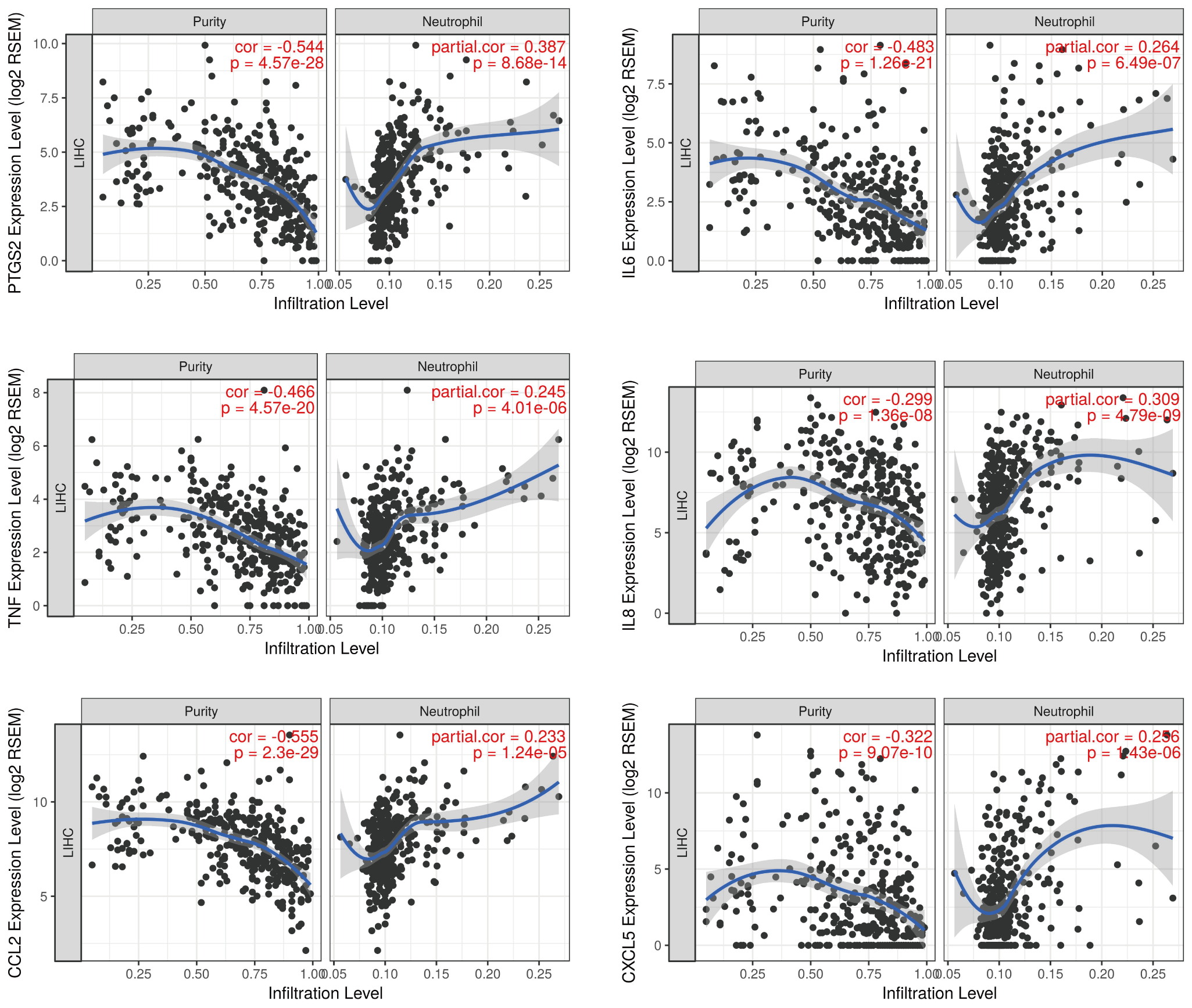

Supplement: Supplementary file 2 — Additional file 2: Figure S1. NETs formation was enhanced in HCC associated neutrophils. Figure S2. Depositions of NETs was correlated with metastasis burden in HCC. Figure S3. NETs in HCC bearing mice. Figure S4. Evidence of NETs formation in TCGA and The Human Protein Atlas database. Figure S5. HCC enhanced the NETs formation capacity of normal neutrophils. Figure S6. NETs formation was enhanced in HCC-driven inflammatory condition in mice. Figure S7. Effect of DNase 1 and neutrophil depletion on LPS-induced NETs in mice. Figure S8. NETs trap optimized adhesion of HCC, and further promoted metastasis potential by raising invasion capacity with minimal cytotoxicity. Figure S9. Effect of DNase 1 on inflammatory mediators expression in NETs-stimulated HCC cells. Figure S10. Correlation analysis of infiltrated neutrophils and COX2 or other inflammatory mediator genes in TIMER database. Figure S11. Pearson correlation analysis of PADI4/MPO and COX2 or other inflammatory mediator genes in TCGA database. Figure S12. Effects of inhibiting IL-1 and TNF-α on NETs-aroused metastasis potential. Figure S13. HCQ impaired the enhanced metastasis potential triggered by NETs. Figure S14. Targeting NETs abrogated tumorous inflammatory response. [file 13045_2019_836_MOESM2_ESM.zip › S-Figure 10.tif]

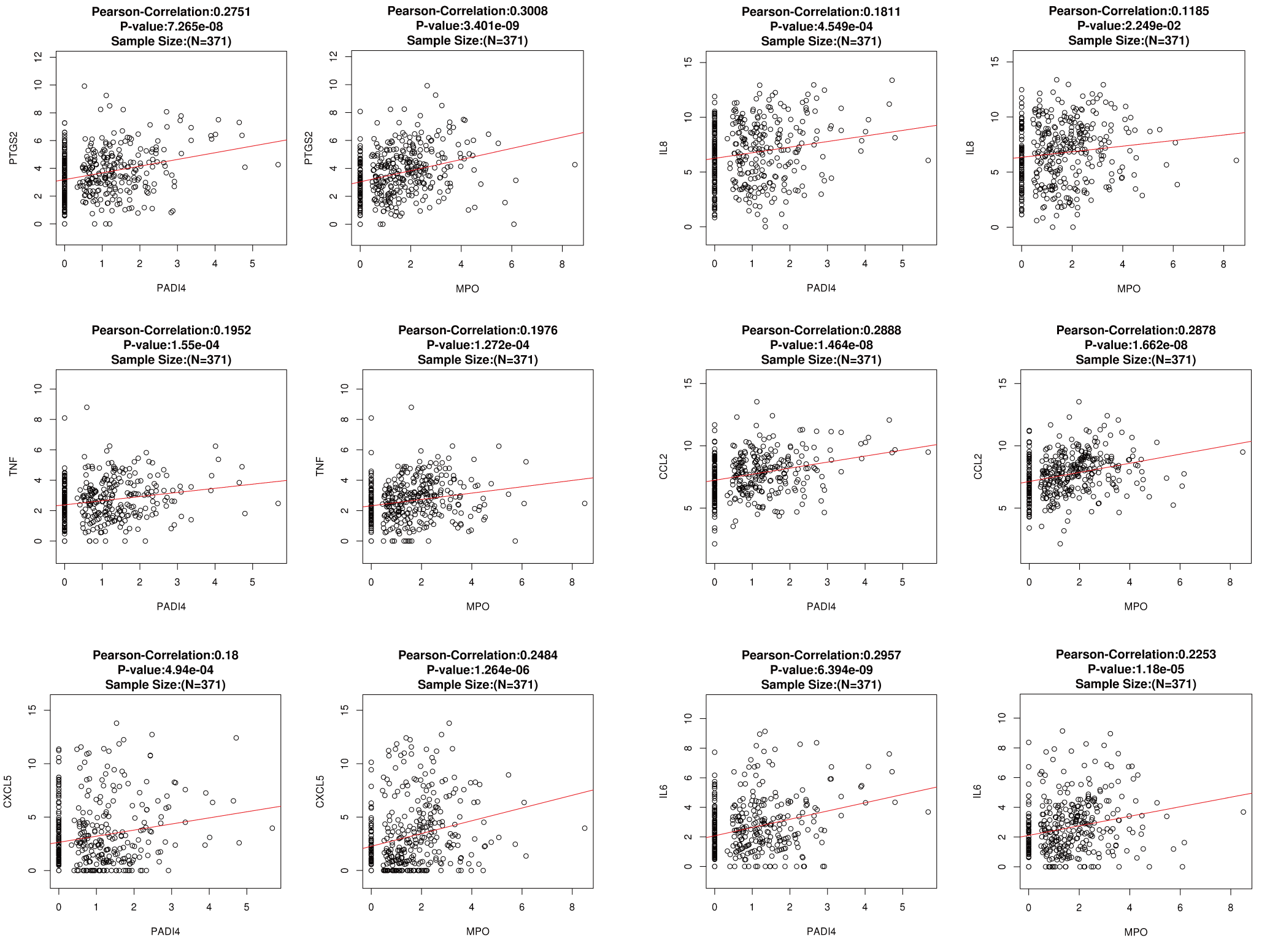

Supplement: Supplementary file 2 — Additional file 2: Figure S1. NETs formation was enhanced in HCC associated neutrophils. Figure S2. Depositions of NETs was correlated with metastasis burden in HCC. Figure S3. NETs in HCC bearing mice. Figure S4. Evidence of NETs formation in TCGA and The Human Protein Atlas database. Figure S5. HCC enhanced the NETs formation capacity of normal neutrophils. Figure S6. NETs formation was enhanced in HCC-driven inflammatory condition in mice. Figure S7. Effect of DNase 1 and neutrophil depletion on LPS-induced NETs in mice. Figure S8. NETs trap optimized adhesion of HCC, and further promoted metastasis potential by raising invasion capacity with minimal cytotoxicity. Figure S9. Effect of DNase 1 on inflammatory mediators expression in NETs-stimulated HCC cells. Figure S10. Correlation analysis of infiltrated neutrophils and COX2 or other inflammatory mediator genes in TIMER database. Figure S11. Pearson correlation analysis of PADI4/MPO and COX2 or other inflammatory mediator genes in TCGA database. Figure S12. Effects of inhibiting IL-1 and TNF-α on NETs-aroused metastasis potential. Figure S13. HCQ impaired the enhanced metastasis potential triggered by NETs. Figure S14. Targeting NETs abrogated tumorous inflammatory response. [file 13045_2019_836_MOESM2_ESM.zip › S-Figure 11.tif]

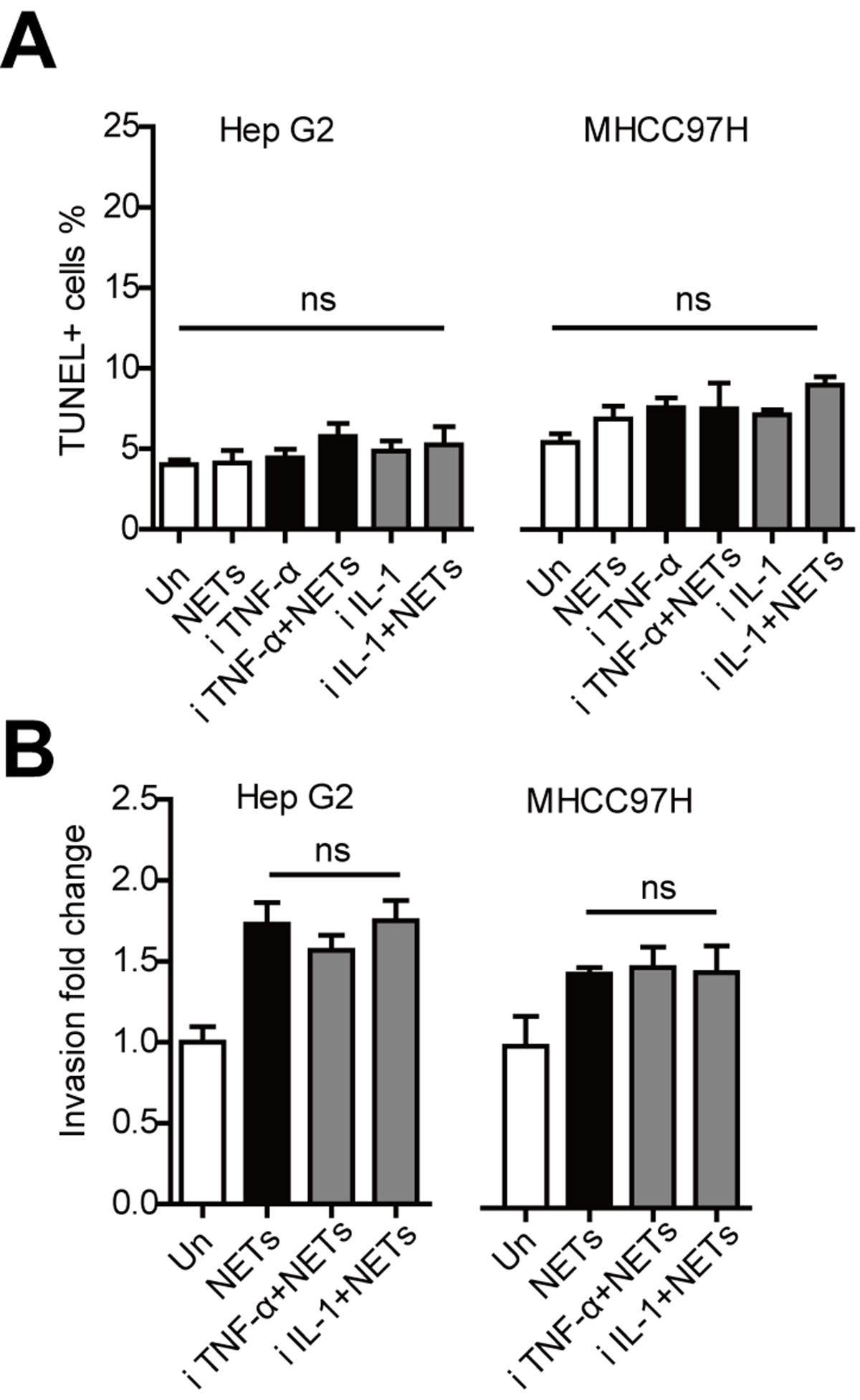

Supplement: Supplementary file 2 — Additional file 2: Figure S1. NETs formation was enhanced in HCC associated neutrophils. Figure S2. Depositions of NETs was correlated with metastasis burden in HCC. Figure S3. NETs in HCC bearing mice. Figure S4. Evidence of NETs formation in TCGA and The Human Protein Atlas database. Figure S5. HCC enhanced the NETs formation capacity of normal neutrophils. Figure S6. NETs formation was enhanced in HCC-driven inflammatory condition in mice. Figure S7. Effect of DNase 1 and neutrophil depletion on LPS-induced NETs in mice. Figure S8. NETs trap optimized adhesion of HCC, and further promoted metastasis potential by raising invasion capacity with minimal cytotoxicity. Figure S9. Effect of DNase 1 on inflammatory mediators expression in NETs-stimulated HCC cells. Figure S10. Correlation analysis of infiltrated neutrophils and COX2 or other inflammatory mediator genes in TIMER database. Figure S11. Pearson correlation analysis of PADI4/MPO and COX2 or other inflammatory mediator genes in TCGA database. Figure S12. Effects of inhibiting IL-1 and TNF-α on NETs-aroused metastasis potential. Figure S13. HCQ impaired the enhanced metastasis potential triggered by NETs. Figure S14. Targeting NETs abrogated tumorous inflammatory response. [file 13045_2019_836_MOESM2_ESM.zip › S-Figure 12.tif]

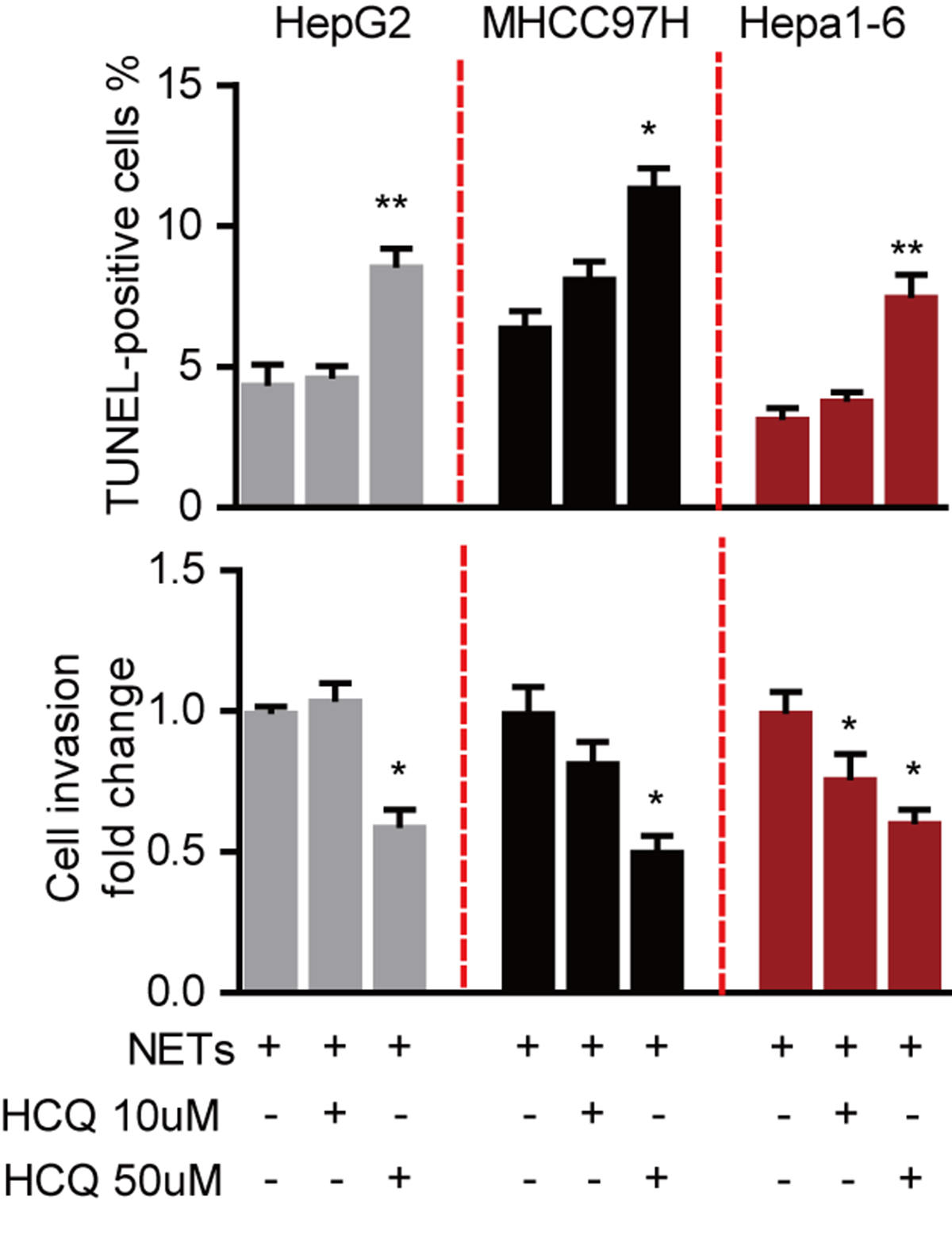

Supplement: Supplementary file 2 — Additional file 2: Figure S1. NETs formation was enhanced in HCC associated neutrophils. Figure S2. Depositions of NETs was correlated with metastasis burden in HCC. Figure S3. NETs in HCC bearing mice. Figure S4. Evidence of NETs formation in TCGA and The Human Protein Atlas database. Figure S5. HCC enhanced the NETs formation capacity of normal neutrophils. Figure S6. NETs formation was enhanced in HCC-driven inflammatory condition in mice. Figure S7. Effect of DNase 1 and neutrophil depletion on LPS-induced NETs in mice. Figure S8. NETs trap optimized adhesion of HCC, and further promoted metastasis potential by raising invasion capacity with minimal cytotoxicity. Figure S9. Effect of DNase 1 on inflammatory mediators expression in NETs-stimulated HCC cells. Figure S10. Correlation analysis of infiltrated neutrophils and COX2 or other inflammatory mediator genes in TIMER database. Figure S11. Pearson correlation analysis of PADI4/MPO and COX2 or other inflammatory mediator genes in TCGA database. Figure S12. Effects of inhibiting IL-1 and TNF-α on NETs-aroused metastasis potential. Figure S13. HCQ impaired the enhanced metastasis potential triggered by NETs. Figure S14. Targeting NETs abrogated tumorous inflammatory response. [file 13045_2019_836_MOESM2_ESM.zip › S-Figure 13.tif]

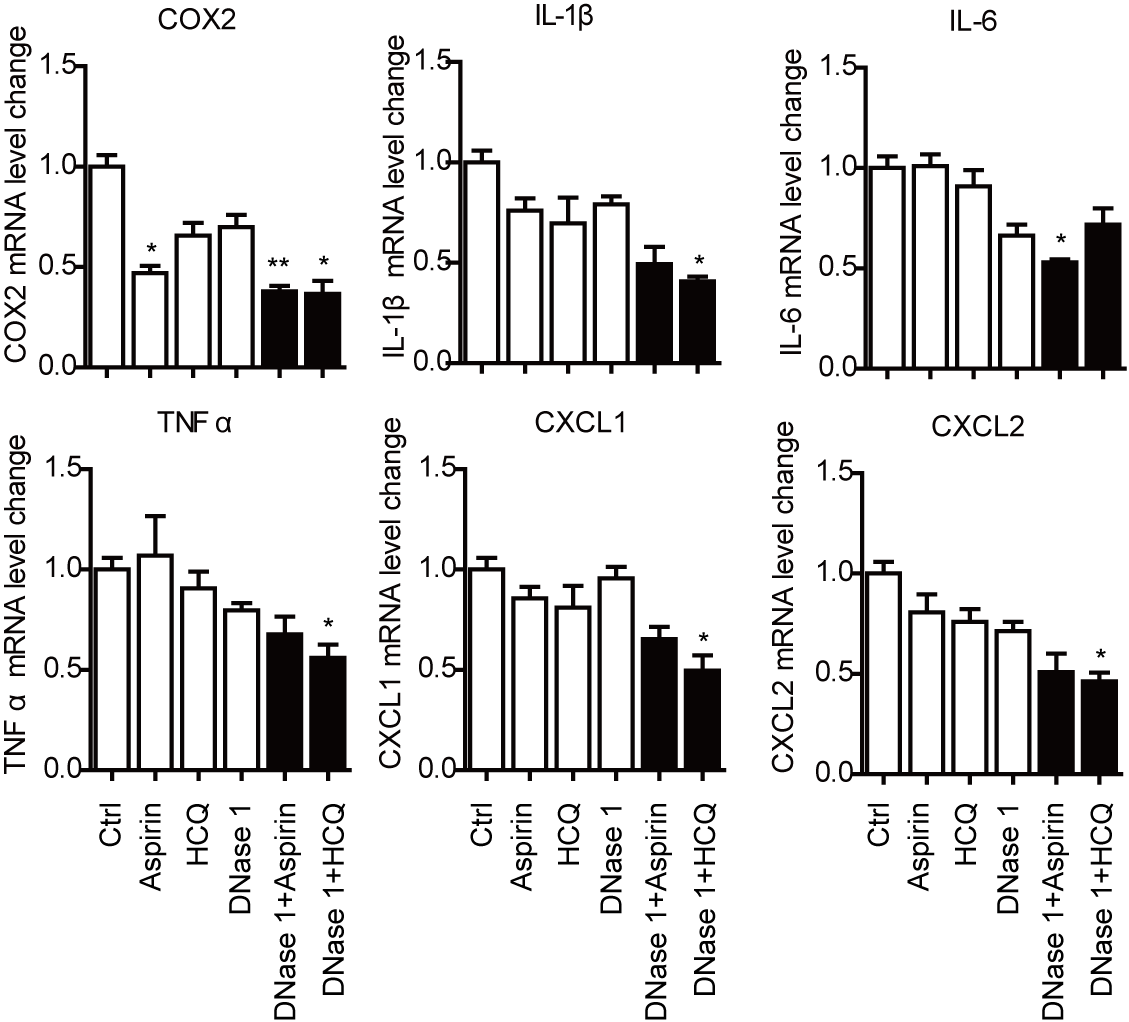

Supplement: Supplementary file 2 — Additional file 2: Figure S1. NETs formation was enhanced in HCC associated neutrophils. Figure S2. Depositions of NETs was correlated with metastasis burden in HCC. Figure S3. NETs in HCC bearing mice. Figure S4. Evidence of NETs formation in TCGA and The Human Protein Atlas database. Figure S5. HCC enhanced the NETs formation capacity of normal neutrophils. Figure S6. NETs formation was enhanced in HCC-driven inflammatory condition in mice. Figure S7. Effect of DNase 1 and neutrophil depletion on LPS-induced NETs in mice. Figure S8. NETs trap optimized adhesion of HCC, and further promoted metastasis potential by raising invasion capacity with minimal cytotoxicity. Figure S9. Effect of DNase 1 on inflammatory mediators expression in NETs-stimulated HCC cells. Figure S10. Correlation analysis of infiltrated neutrophils and COX2 or other inflammatory mediator genes in TIMER database. Figure S11. Pearson correlation analysis of PADI4/MPO and COX2 or other inflammatory mediator genes in TCGA database. Figure S12. Effects of inhibiting IL-1 and TNF-α on NETs-aroused metastasis potential. Figure S13. HCQ impaired the enhanced metastasis potential triggered by NETs. Figure S14. Targeting NETs abrogated tumorous inflammatory response. [file 13045_2019_836_MOESM2_ESM.zip › S-Figure 14.tif]

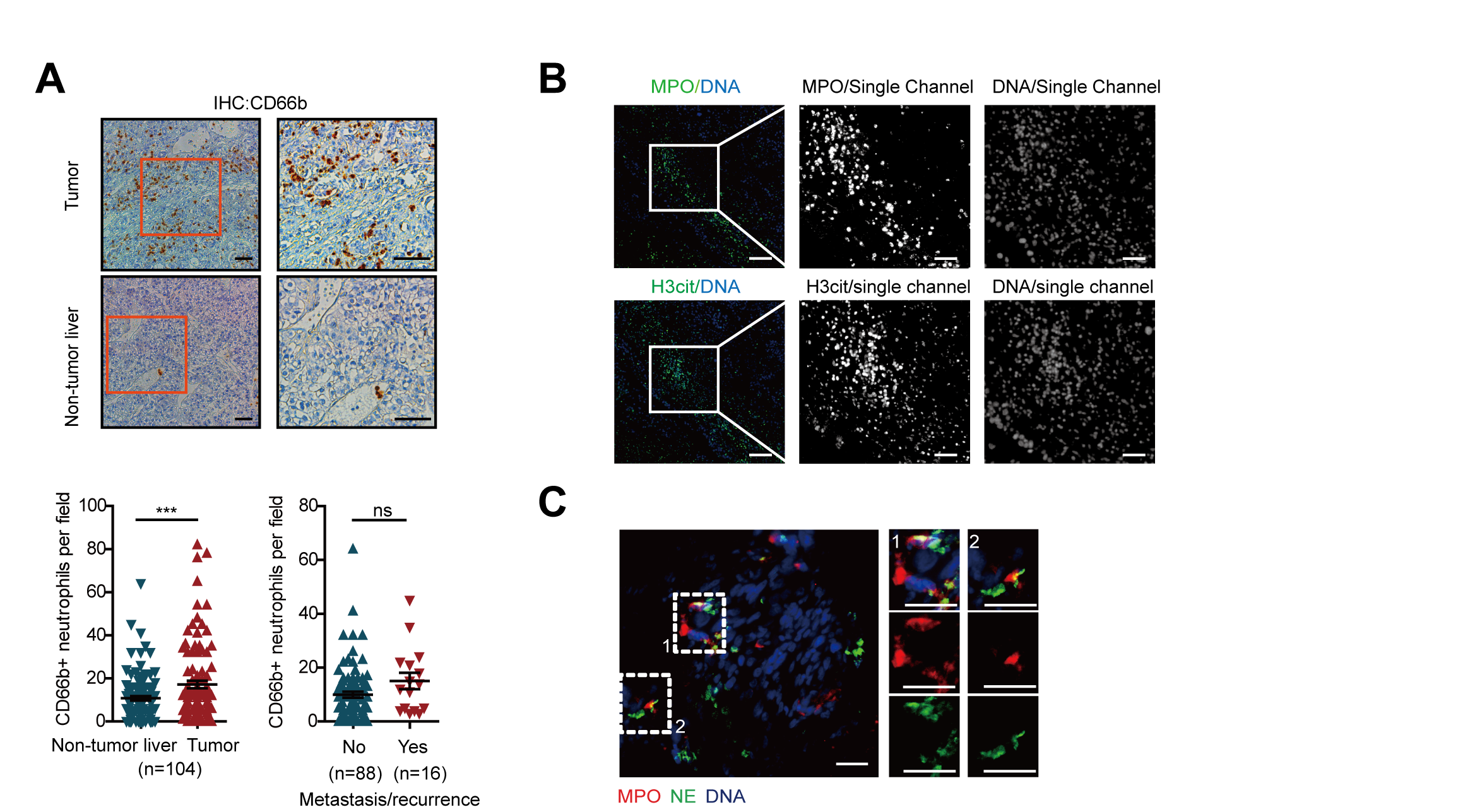

Supplement: Supplementary file 2 — Additional file 2: Figure S1. NETs formation was enhanced in HCC associated neutrophils. Figure S2. Depositions of NETs was correlated with metastasis burden in HCC. Figure S3. NETs in HCC bearing mice. Figure S4. Evidence of NETs formation in TCGA and The Human Protein Atlas database. Figure S5. HCC enhanced the NETs formation capacity of normal neutrophils. Figure S6. NETs formation was enhanced in HCC-driven inflammatory condition in mice. Figure S7. Effect of DNase 1 and neutrophil depletion on LPS-induced NETs in mice. Figure S8. NETs trap optimized adhesion of HCC, and further promoted metastasis potential by raising invasion capacity with minimal cytotoxicity. Figure S9. Effect of DNase 1 on inflammatory mediators expression in NETs-stimulated HCC cells. Figure S10. Correlation analysis of infiltrated neutrophils and COX2 or other inflammatory mediator genes in TIMER database. Figure S11. Pearson correlation analysis of PADI4/MPO and COX2 or other inflammatory mediator genes in TCGA database. Figure S12. Effects of inhibiting IL-1 and TNF-α on NETs-aroused metastasis potential. Figure S13. HCQ impaired the enhanced metastasis potential triggered by NETs. Figure S14. Targeting NETs abrogated tumorous inflammatory response. [file 13045_2019_836_MOESM2_ESM.zip › S-Figure 2.tif]

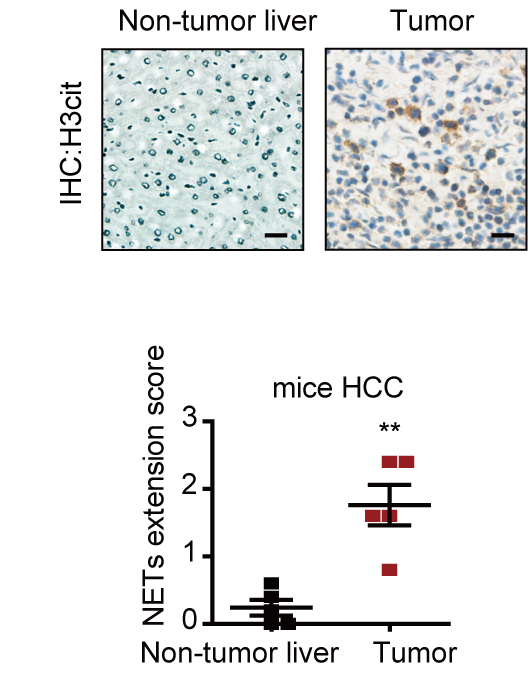

Supplement: Supplementary file 2 — Additional file 2: Figure S1. NETs formation was enhanced in HCC associated neutrophils. Figure S2. Depositions of NETs was correlated with metastasis burden in HCC. Figure S3. NETs in HCC bearing mice. Figure S4. Evidence of NETs formation in TCGA and The Human Protein Atlas database. Figure S5. HCC enhanced the NETs formation capacity of normal neutrophils. Figure S6. NETs formation was enhanced in HCC-driven inflammatory condition in mice. Figure S7. Effect of DNase 1 and neutrophil depletion on LPS-induced NETs in mice. Figure S8. NETs trap optimized adhesion of HCC, and further promoted metastasis potential by raising invasion capacity with minimal cytotoxicity. Figure S9. Effect of DNase 1 on inflammatory mediators expression in NETs-stimulated HCC cells. Figure S10. Correlation analysis of infiltrated neutrophils and COX2 or other inflammatory mediator genes in TIMER database. Figure S11. Pearson correlation analysis of PADI4/MPO and COX2 or other inflammatory mediator genes in TCGA database. Figure S12. Effects of inhibiting IL-1 and TNF-α on NETs-aroused metastasis potential. Figure S13. HCQ impaired the enhanced metastasis potential triggered by NETs. Figure S14. Targeting NETs abrogated tumorous inflammatory response. [file 13045_2019_836_MOESM2_ESM.zip › S-Figure 3.tif]

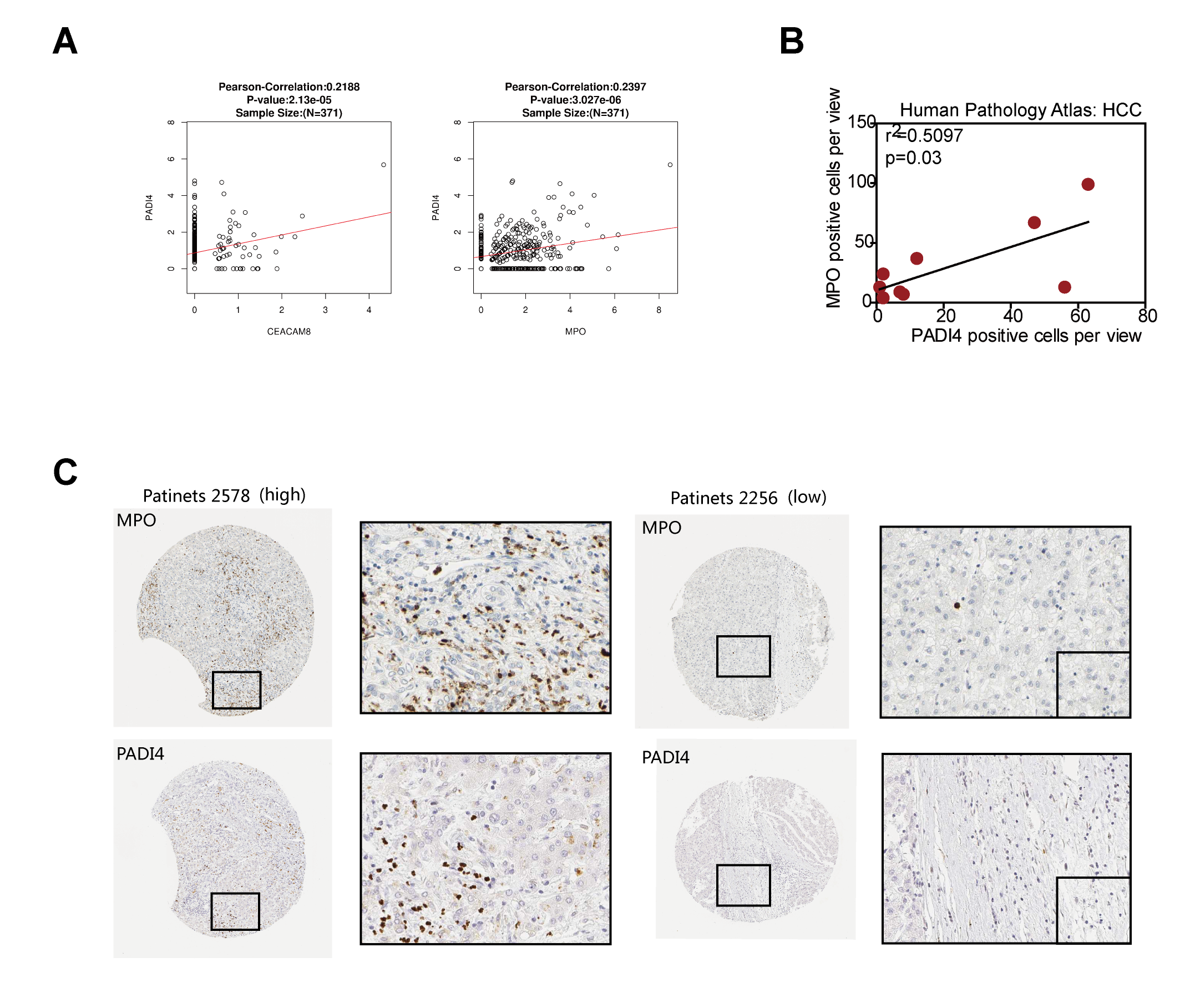

Supplement: Supplementary file 2 — Additional file 2: Figure S1. NETs formation was enhanced in HCC associated neutrophils. Figure S2. Depositions of NETs was correlated with metastasis burden in HCC. Figure S3. NETs in HCC bearing mice. Figure S4. Evidence of NETs formation in TCGA and The Human Protein Atlas database. Figure S5. HCC enhanced the NETs formation capacity of normal neutrophils. Figure S6. NETs formation was enhanced in HCC-driven inflammatory condition in mice. Figure S7. Effect of DNase 1 and neutrophil depletion on LPS-induced NETs in mice. Figure S8. NETs trap optimized adhesion of HCC, and further promoted metastasis potential by raising invasion capacity with minimal cytotoxicity. Figure S9. Effect of DNase 1 on inflammatory mediators expression in NETs-stimulated HCC cells. Figure S10. Correlation analysis of infiltrated neutrophils and COX2 or other inflammatory mediator genes in TIMER database. Figure S11. Pearson correlation analysis of PADI4/MPO and COX2 or other inflammatory mediator genes in TCGA database. Figure S12. Effects of inhibiting IL-1 and TNF-α on NETs-aroused metastasis potential. Figure S13. HCQ impaired the enhanced metastasis potential triggered by NETs. Figure S14. Targeting NETs abrogated tumorous inflammatory response. [file 13045_2019_836_MOESM2_ESM.zip › S-Figure 4.tif]

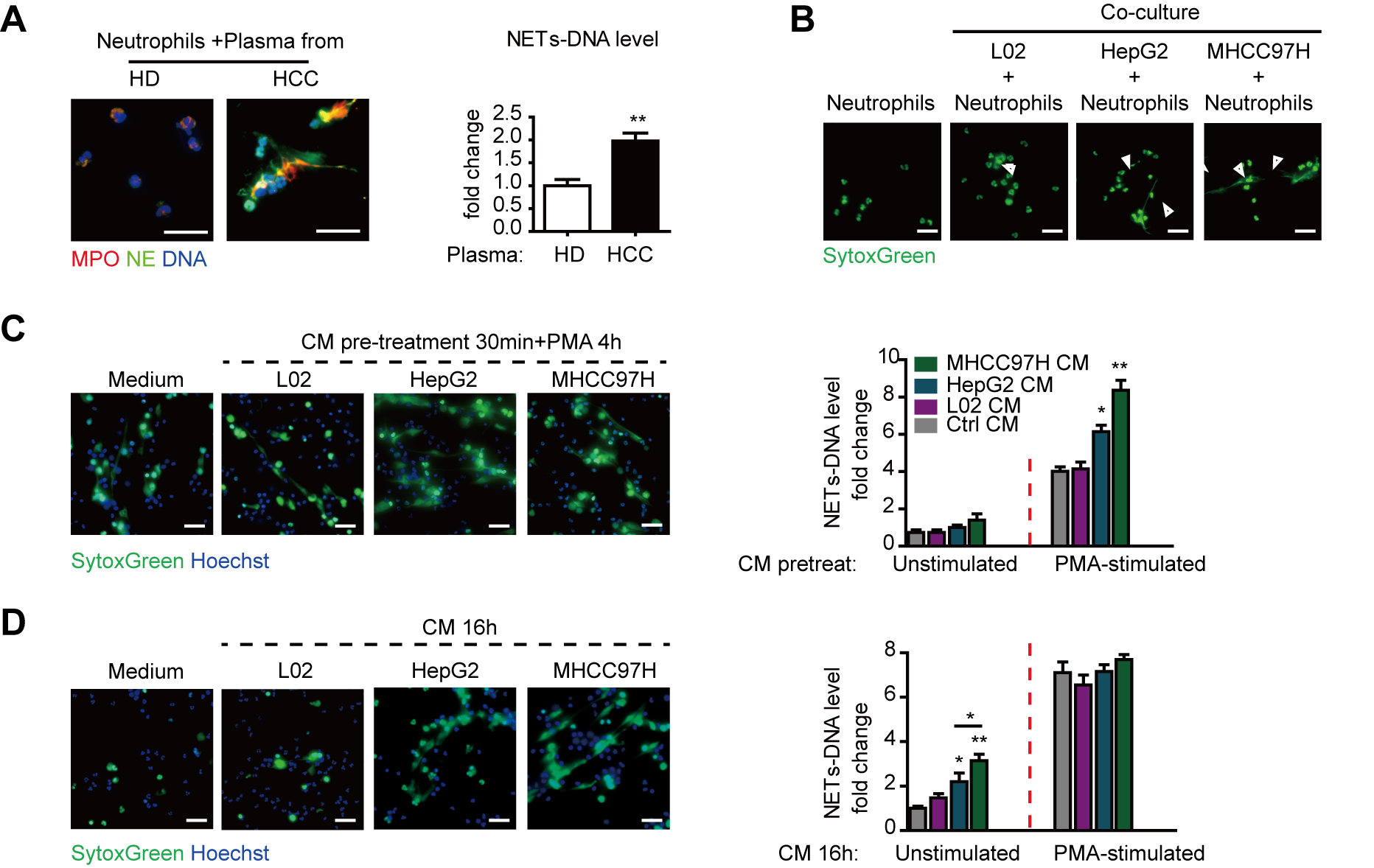

Supplement: Supplementary file 2 — Additional file 2: Figure S1. NETs formation was enhanced in HCC associated neutrophils. Figure S2. Depositions of NETs was correlated with metastasis burden in HCC. Figure S3. NETs in HCC bearing mice. Figure S4. Evidence of NETs formation in TCGA and The Human Protein Atlas database. Figure S5. HCC enhanced the NETs formation capacity of normal neutrophils. Figure S6. NETs formation was enhanced in HCC-driven inflammatory condition in mice. Figure S7. Effect of DNase 1 and neutrophil depletion on LPS-induced NETs in mice. Figure S8. NETs trap optimized adhesion of HCC, and further promoted metastasis potential by raising invasion capacity with minimal cytotoxicity. Figure S9. Effect of DNase 1 on inflammatory mediators expression in NETs-stimulated HCC cells. Figure S10. Correlation analysis of infiltrated neutrophils and COX2 or other inflammatory mediator genes in TIMER database. Figure S11. Pearson correlation analysis of PADI4/MPO and COX2 or other inflammatory mediator genes in TCGA database. Figure S12. Effects of inhibiting IL-1 and TNF-α on NETs-aroused metastasis potential. Figure S13. HCQ impaired the enhanced metastasis potential triggered by NETs. Figure S14. Targeting NETs abrogated tumorous inflammatory response. [file 13045_2019_836_MOESM2_ESM.zip › S-Figure 5.tif]

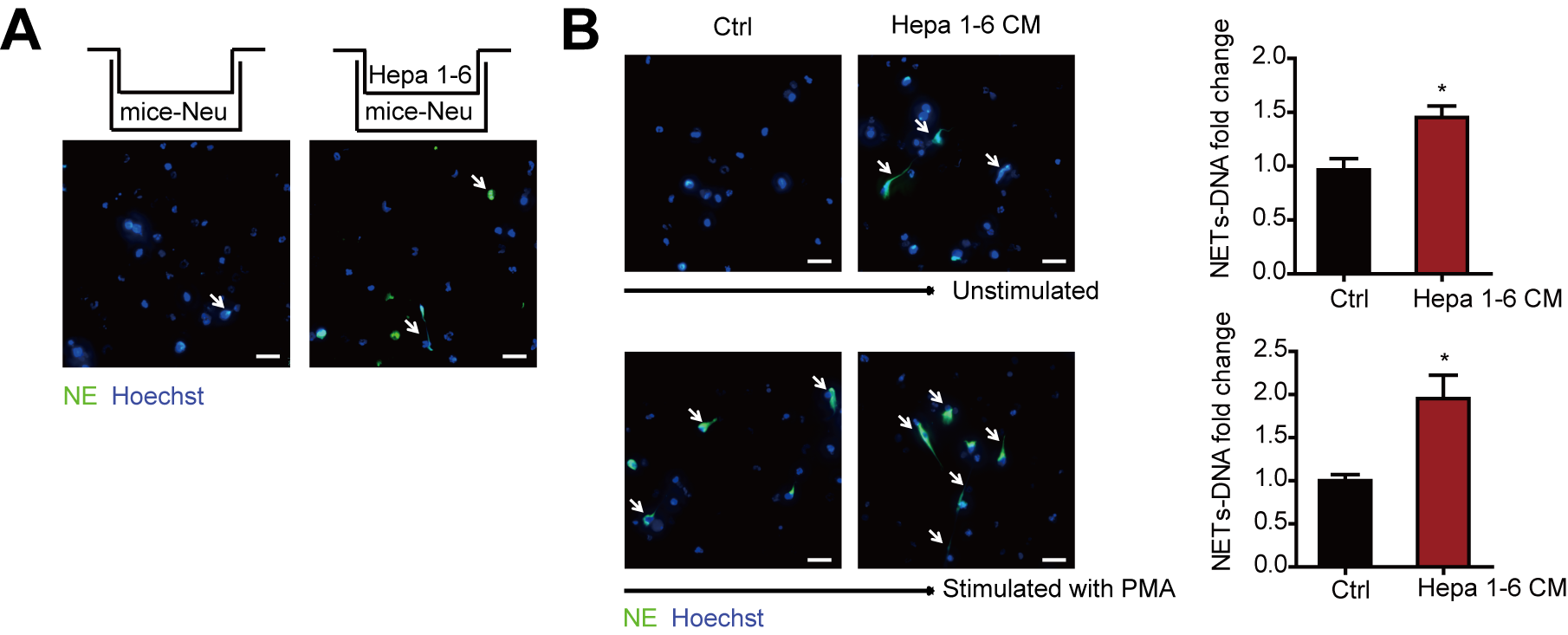

Supplement: Supplementary file 2 — Additional file 2: Figure S1. NETs formation was enhanced in HCC associated neutrophils. Figure S2. Depositions of NETs was correlated with metastasis burden in HCC. Figure S3. NETs in HCC bearing mice. Figure S4. Evidence of NETs formation in TCGA and The Human Protein Atlas database. Figure S5. HCC enhanced the NETs formation capacity of normal neutrophils. Figure S6. NETs formation was enhanced in HCC-driven inflammatory condition in mice. Figure S7. Effect of DNase 1 and neutrophil depletion on LPS-induced NETs in mice. Figure S8. NETs trap optimized adhesion of HCC, and further promoted metastasis potential by raising invasion capacity with minimal cytotoxicity. Figure S9. Effect of DNase 1 on inflammatory mediators expression in NETs-stimulated HCC cells. Figure S10. Correlation analysis of infiltrated neutrophils and COX2 or other inflammatory mediator genes in TIMER database. Figure S11. Pearson correlation analysis of PADI4/MPO and COX2 or other inflammatory mediator genes in TCGA database. Figure S12. Effects of inhibiting IL-1 and TNF-α on NETs-aroused metastasis potential. Figure S13. HCQ impaired the enhanced metastasis potential triggered by NETs. Figure S14. Targeting NETs abrogated tumorous inflammatory response. [file 13045_2019_836_MOESM2_ESM.zip › S-Figure 6.tif]

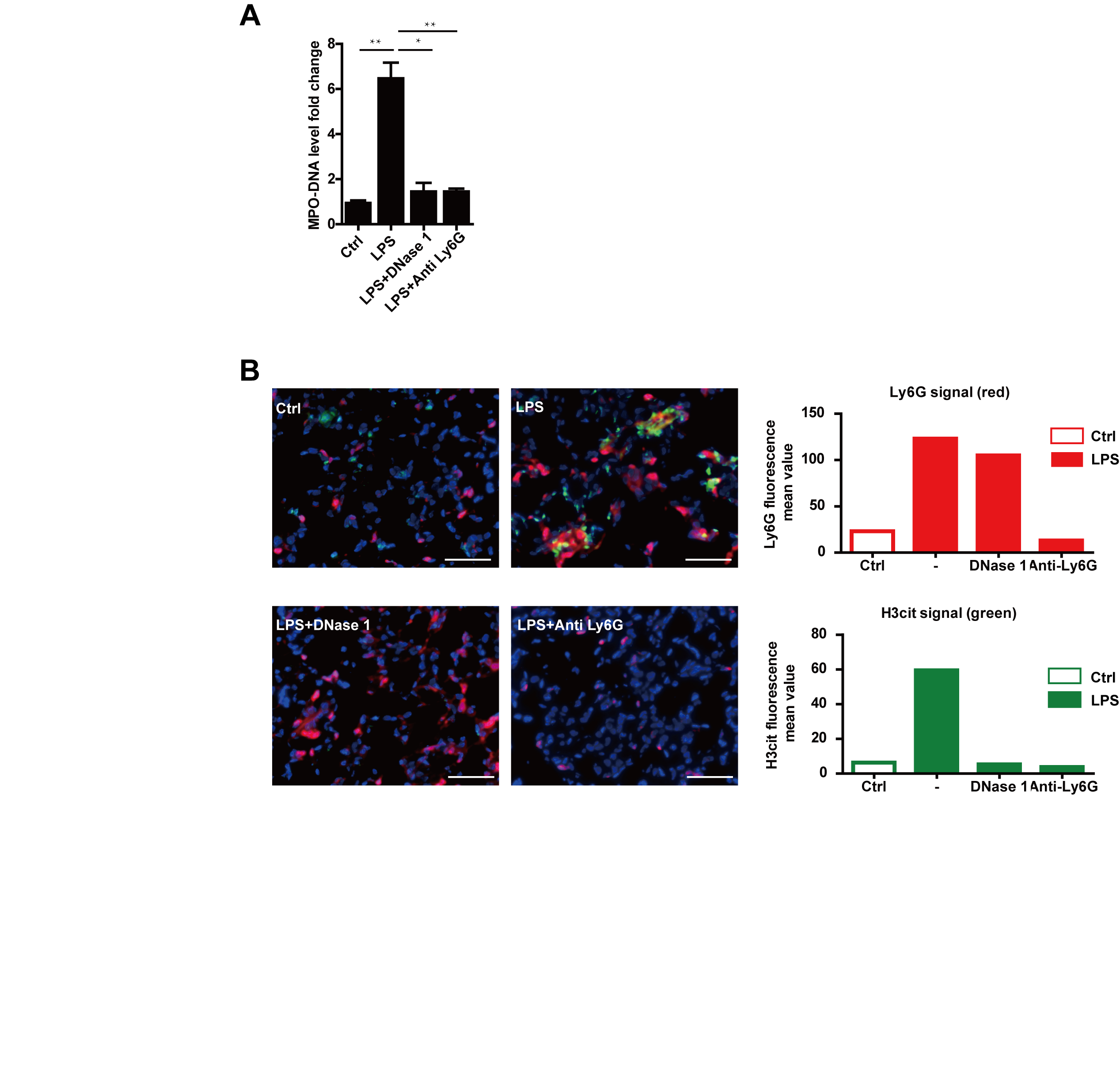

Supplement: Supplementary file 2 — Additional file 2: Figure S1. NETs formation was enhanced in HCC associated neutrophils. Figure S2. Depositions of NETs was correlated with metastasis burden in HCC. Figure S3. NETs in HCC bearing mice. Figure S4. Evidence of NETs formation in TCGA and The Human Protein Atlas database. Figure S5. HCC enhanced the NETs formation capacity of normal neutrophils. Figure S6. NETs formation was enhanced in HCC-driven inflammatory condition in mice. Figure S7. Effect of DNase 1 and neutrophil depletion on LPS-induced NETs in mice. Figure S8. NETs trap optimized adhesion of HCC, and further promoted metastasis potential by raising invasion capacity with minimal cytotoxicity. Figure S9. Effect of DNase 1 on inflammatory mediators expression in NETs-stimulated HCC cells. Figure S10. Correlation analysis of infiltrated neutrophils and COX2 or other inflammatory mediator genes in TIMER database. Figure S11. Pearson correlation analysis of PADI4/MPO and COX2 or other inflammatory mediator genes in TCGA database. Figure S12. Effects of inhibiting IL-1 and TNF-α on NETs-aroused metastasis potential. Figure S13. HCQ impaired the enhanced metastasis potential triggered by NETs. Figure S14. Targeting NETs abrogated tumorous inflammatory response. [file 13045_2019_836_MOESM2_ESM.zip › S-Figure 7.tif]

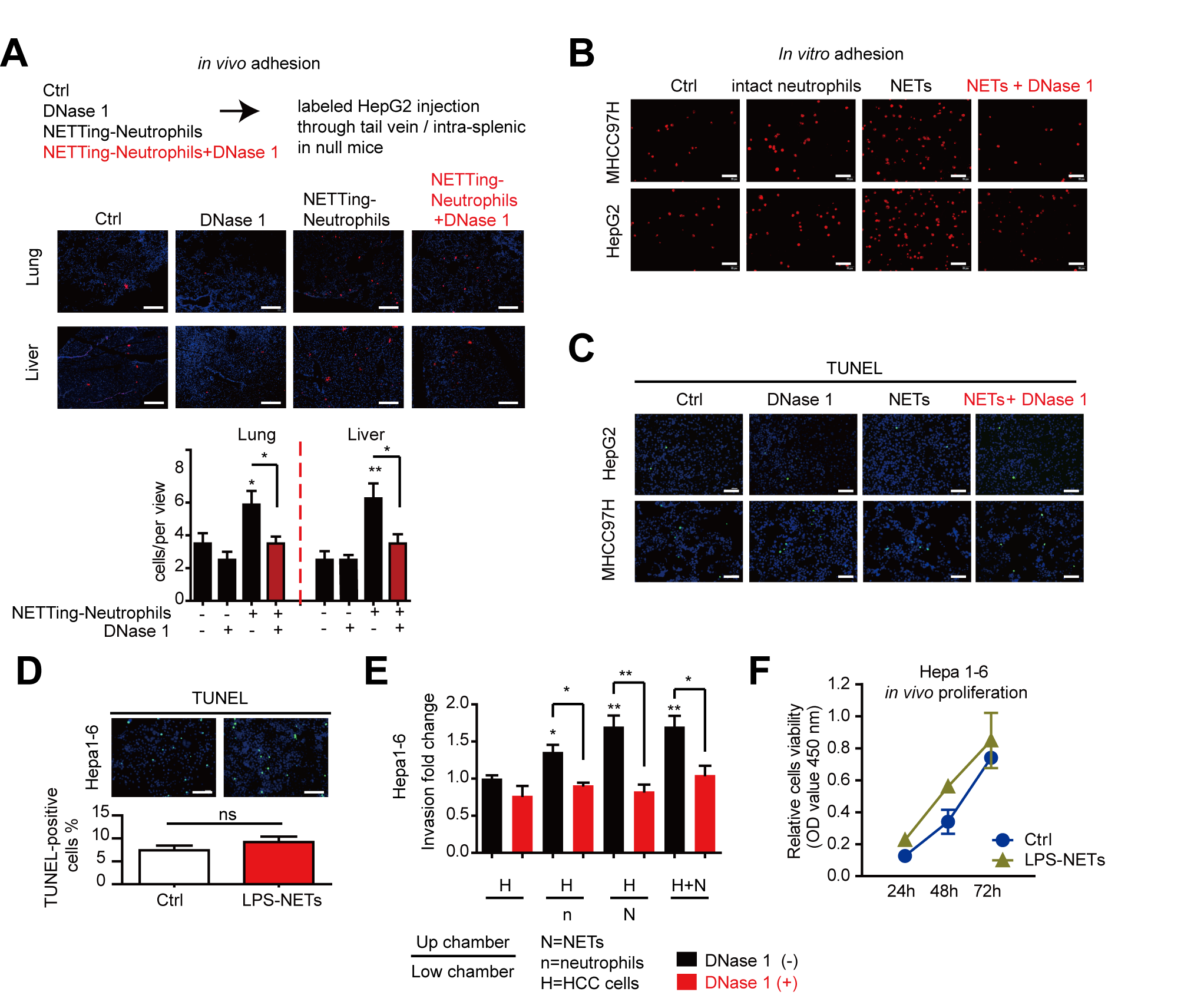

Supplement: Supplementary file 2 — Additional file 2: Figure S1. NETs formation was enhanced in HCC associated neutrophils. Figure S2. Depositions of NETs was correlated with metastasis burden in HCC. Figure S3. NETs in HCC bearing mice. Figure S4. Evidence of NETs formation in TCGA and The Human Protein Atlas database. Figure S5. HCC enhanced the NETs formation capacity of normal neutrophils. Figure S6. NETs formation was enhanced in HCC-driven inflammatory condition in mice. Figure S7. Effect of DNase 1 and neutrophil depletion on LPS-induced NETs in mice. Figure S8. NETs trap optimized adhesion of HCC, and further promoted metastasis potential by raising invasion capacity with minimal cytotoxicity. Figure S9. Effect of DNase 1 on inflammatory mediators expression in NETs-stimulated HCC cells. Figure S10. Correlation analysis of infiltrated neutrophils and COX2 or other inflammatory mediator genes in TIMER database. Figure S11. Pearson correlation analysis of PADI4/MPO and COX2 or other inflammatory mediator genes in TCGA database. Figure S12. Effects of inhibiting IL-1 and TNF-α on NETs-aroused metastasis potential. Figure S13. HCQ impaired the enhanced metastasis potential triggered by NETs. Figure S14. Targeting NETs abrogated tumorous inflammatory response. [file 13045_2019_836_MOESM2_ESM.zip › S-Figure 8.tif]

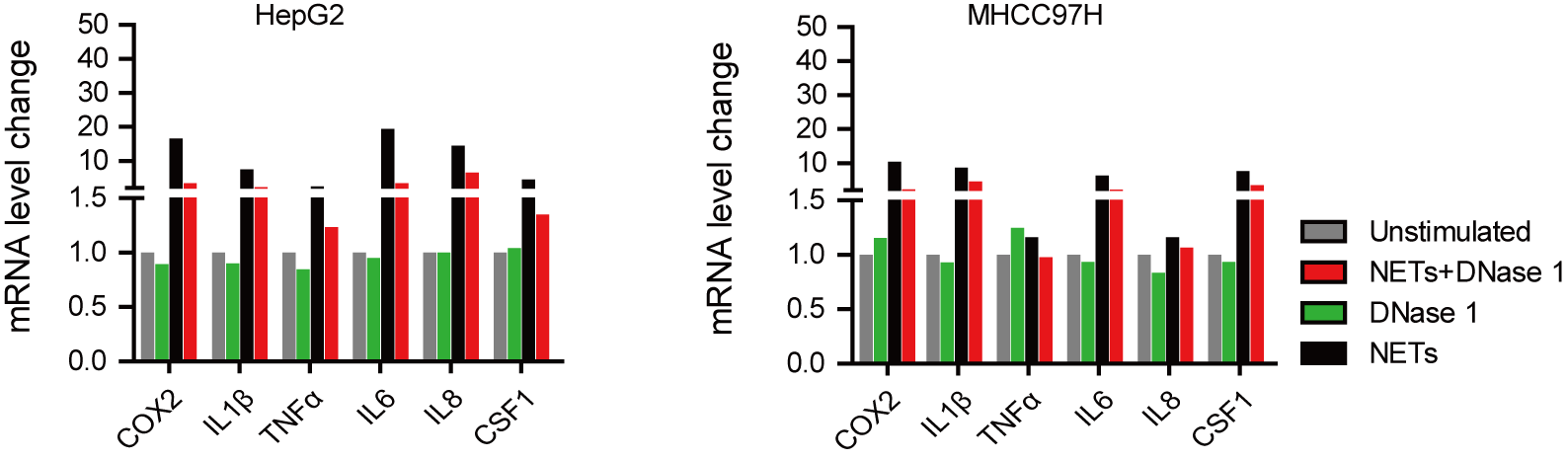

Supplement: Supplementary file 2 — Additional file 2: Figure S1. NETs formation was enhanced in HCC associated neutrophils. Figure S2. Depositions of NETs was correlated with metastasis burden in HCC. Figure S3. NETs in HCC bearing mice. Figure S4. Evidence of NETs formation in TCGA and The Human Protein Atlas database. Figure S5. HCC enhanced the NETs formation capacity of normal neutrophils. Figure S6. NETs formation was enhanced in HCC-driven inflammatory condition in mice. Figure S7. Effect of DNase 1 and neutrophil depletion on LPS-induced NETs in mice. Figure S8. NETs trap optimized adhesion of HCC, and further promoted metastasis potential by raising invasion capacity with minimal cytotoxicity. Figure S9. Effect of DNase 1 on inflammatory mediators expression in NETs-stimulated HCC cells. Figure S10. Correlation analysis of infiltrated neutrophils and COX2 or other inflammatory mediator genes in TIMER database. Figure S11. Pearson correlation analysis of PADI4/MPO and COX2 or other inflammatory mediator genes in TCGA database. Figure S12. Effects of inhibiting IL-1 and TNF-α on NETs-aroused metastasis potential. Figure S13. HCQ impaired the enhanced metastasis potential triggered by NETs. Figure S14. Targeting NETs abrogated tumorous inflammatory response. [file 13045_2019_836_MOESM2_ESM.zip › S-Figure 9.tif]
