# Supplementary material for: Increased neutrophil extracellular traps promote metastasis potential of hepatocellular carcinoma via provoking tumorous inflammatory response
Source: J Hematol Oncol. 2020 Jan 6;13:3. doi: 10.1186/s13045-019-0836-0 (PMC6945602; doi:10.1186/s13045-019-0836-0)
Supplement: Supplementary file 3 — Additional file 3: Table S1. Clinicopathological characteristics of HCC patients for NETs pathological analysis (n = 104). Table S2. Clinicopathological characteristics of HCC patients for serum MPO-DNA detection (n = 73). Table S3. Real-time PCR primers used in the study. Table S4. Primary antibodies used in the study. [file 13045_2019_836_MOESM3_ESM.docx]

| **Table S1. Clinicopathological characteristics of HCC patients for NETs pathological analysis (n = 104)** | | | | |
| --- | --- | --- | --- | --- |
|  |  |  |  |  |
|  | Characteristics | Case number | % |  |
|  |  |  |  |  |
|  | Metastasis/recurrence |  |  |  |
|  | No | 88 | 84.6% |  |
|  | Yes | 16 | 15.4% |  |
|  | Sex |  |  |  |
|  | Female | 21 | 20.2% |  |
|  | Male | 83 | 79.8% |  |
|  | Age (years) |  |  |  |
|  | ≤ 55 | 27 | 26.0% |  |
|  | > 55 | 77 | 74.0% |  |
|  | Preoperative serum AFP (ng/mL) |  |  |  |
|  | ≤ 20 | 19 | 18.3% |  |
|  | > 20 | 85 | 81.7% |  |
|  | HBsAg |  |  |  |
|  | Negative | 33 | 31.7% |  |
|  | Positive | 71 | 68.3% |  |
|  | Liver cirrhosis |  |  |  |
|  | No | 46 | 44.2% |  |
|  | Yes | 58 | 55.8% |  |
|  | Tumor size |  |  |  |
|  | ≤ 5cm | 68 | 65.4% |  |
|  | > 5cm | 36 | 34.6% |  |
|  | Tumor number |  |  |  |
|  | Single | 82 | 78.8% |  |
|  | Multiple | 22 | 21.2% |  |
|  | Tumor encapsulation |  |  |  |
|  | None | 45 | 43.3% |  |
|  | Complete | 59 | 56.7% |  |
|  | Vascular invasion |  |  |  |
|  | No | 63 | 60.6% |  |
|  | Yes | 41 | 39.4% |  |

| **Table S2. Clinicopathological characteristics of HCC patients for serum MPO-DNA detection (n = 73)** | | | | | |
| --- | --- | --- | --- | --- | --- |
|  |  |  |  |  |  |
|  | Characteristics | Case number | % |  |  |
|  |  |  |  |  |  |
|  | Metastasis/recurrence |  |  |  |  |
|  | No | 50 | 68.5% |  |  |
|  | Pulmonary | 7 | 9.6% |  |  |
|  | Intrahepatic | 16 | 21.9% |  |  |
|  | Sex |  |  |  |  |
|  | Female | 20 | 27.4% |  |  |
|  | Male | 53 | 72.6% |  |  |
|  | Age (years) |  |  |  |  |
|  | ≤ 55 | 23 | 31.5% |  |  |
|  | > 55 | 50 | 68.5% |  |  |
|  | Preoperative serum AFP (ng/mL) |  |  |  |  |
|  | ≤ 20 | 12 | 16.4% |  |  |
|  | > 20 | 61 | 83.6% |  |  |
|  | HBsAg |  |  |  |  |
|  | Negative | 19 | 26.0% |  |  |
|  | Positive | 54 | 74.0% |  |  |
|  | Tumor size |  |  |  |  |
|  | ≤ 5cm | 43 | 58.9% |  |  |
|  | > 5cm | 30 | 41.1% |  |  |
|  | Tumor number |  |  |  |  |
|  | Single | 43 | 58.9% |  |  |
|  | Multiple | 30 | 41.1% |  |  |

|  |  |  |
| --- | --- | --- |
| \| **Table S3. Real-time PCR primers used in the study** \| \| \| --- \| --- \| \| Primers \| Seqeunces(5’-3’) \| \| Human \|  \| \| TLR1-(forward) \| CCACGTTCCTAAAGACCTATCCC \| \| TLR1-(reverse) \| CCAAGTGCTTGAGGTTCACAG \| \| TLR2-(forward) \| ATCCTCCAATCAGGCTTCTCT \| \| TLR2-(reverse) \| GGACAGGTCAAGGCTTTTTACA \| \| TLR3-(forward) \| TTGCCTTGTATCTACTTTTGGGG \| \| TLR3-(reverse) \| TCAACACTGTTATGTTTGTGGGT \| \| TLR4-(forward) \| AGACCTGTCCCTGAACCCTAT \| \| TLR4-(reverse) \| CGATGGACTTCTAAACCAGCCA \| \| TLR5-(forward) \| GCCGGTCCTGTGTTTGGAAT \| \| TLR5-(reverse) \| GGTGAGGTTGCAGAAACGATAAA \| \| TLR6-(forward) \| TTCTCCGACGGAAATGAATTTGC \| \| TLR6-(reverse) \| CAGCGGTAGGTCTTTTGGAAC \| \| TLR7-(forward) \| TCCTTGGGGCTAGATGGTTTC \| \| TLR7-(reverse) \| TCCACGATCACATGGTTCTTTG \| \| TLR8-(forward) \| ATGTTCCTTCAGTCGTCAATGC \| \| TLR8-(reverse) \| TTGCTGCACTCTGCAATAACT \| \| TLR9-(forward) \| CTGCCTTCCTACCCTGTGAG \| \| TLR9-(reverse) \| GGATGCGGTTGGAGGACAA \| \| TLR10-(forward) \| AGGTTTGAGTGGGGCAAAAAT \| \| TLR10-(reverse) \| CCATCACGCAAAAGAACCCAG \| \| AIM2-(forward) \| TGGCAAAACGTCTTCAGGAGG \| \| AIM2-(reverse) \| AGCTTGACTTAGTGGCTTTGG \| \| STING-(forward) \| CCAGAGCACACTCTCCGGTA \| \| STING-(reverse) \| CGCATTTGGGAGGGAGTAGTA \| \| DAI-(forward) \| AACATGCAGCTACAATTCCAGA \| \| DAI-(reverse) \| AGTCTCGGTTCACATCTTTTGC \| \| nod2-(forward) \| TGGTTCAGCCTCTCACGATGA \| \| nod2-(reverse) \| CAGGACACTCTCGAAGCCTT \| \| NLRP3-(forward) \| GATCTTCGCTGCGATCAACAG \| \| NLRP3-(reverse) \| CGTGCATTATCTGAACCCCAC \| \| RAGE-(forward) \| GTGTCCTTCCCAACGGCTC \| \| RAGE-(reverse) \| ATTGCCTGGCACCGGAAAA \| \| Cox2-(forward) \| GAGAAAACTGCTCAACACCG \| \| Cox2-(reverse) \| GCATACTCTGTTGTGTTCCC \| \| IL-8-(forward)  IL-8-(reverse)  IL-6-(forward)  IL-6-(reverse)  IL-1β-(forward)  IL-1β-(reverse)  TNFα-(forward)  TNFα-(reverse)  CSF1-(forward)  CSF1-(reverse)  cGAS-(forward) \| CTCCAGCCACACTCCAACAGA  CACCCTAACACAAAACACGAT  CCACGGCCTTCCCTACTTC  CTGTTGGGAGTGGTATCCTCTGT  CTAAAGTATGGGCTGGACTG  AGCTTCAATGAAAGACCTCA  GACGTGGAACTGGCAGAAGAG  TCGGACAAGCAGGAATGAGA  TCCAGCCAAGATGTGGTGAC  AGTTCCCTCAGAGTCCTCCC  CACGAAGCCAAGACCTCCG \| \| cGAS-(reverse) \| GTCGCACTTCAGTCTGAGCA \| \| mouse \|  \| \| COX2-(forward) \| ACCAGCAGTTCCAGTATCAG \| \| COX2-(reverse) \| AGGAGGATGGAGTTGTTGTAG \| \| IL-1β-(forward) \| AGCTTCAGGCAGGCAGTATC \| \| IL-1β-(reverse) \| CGTCACACACCAGCAGGTTA \| \| IL-6-(forward) \| GAGGATACCACTCCCAACAGACC \| \| IL-6-(reverse) \| AAGTGCATCATCGTTGTTCATACA \| \| CXCL1-(forward) \| CCGAAGTCATAGCCACACTCAA \| \| CXCL1-(reverse) \| GCAGTCTGTCTTCTTTCTCCGTTA \| \| CXCL2-(forward) \| GAAGTCATAGCCACTCTCAAGG \| \| CXCL2-(reverse) \| CCTCCTTTCCAGGTCAGTTAGC \| \| TNFα-(forward) \| AGCCCCCAGTCTGTATCCTT \| \| TNFα-(reverse) \| CTCCCTTTGCAGAACTCAGG \| | |  |

| **Table S4. Primary antibodies used in the study** | | | | | | |  |
| --- | --- | --- | --- | --- | --- | --- | --- |
| Primary antibody | For WB | For IHC | For IF | For ELISA | Specificity | Company |  |
| H3cit | 1:300 | 1:250 | 1:200 | \ | Rabbit polyclonal | Abcam |  |
| CD66b | \ | 1:200 | \ | \ | Mouse monoclonal | BD |  |
| MPO (Human) | \ | \ | 1:100 | 1:100 | Mouse monoclonal | Abcam |  |
| MPO (mouse) | \ | \ | \ | 1:100 | Rabbit monoclonal | Santa Cruz |  |
| NE | \ | \ | 1:150 | \ | Rabbit polyclonal | Abcam |  |
| Cox2 | 1:250 | 1:150 | 1:100 | \ | Rabbit polyclonal | Abcam |  |
| TLR4 | 1:400 | \ | \ | \ | mouse monoclonal | Santa Cruz |  |
| TLR9 | 1:400 | \ | \ | \ | mouse monoclonal | Santa Cruz |  |
| Akt /P-Akt | 1:500 | \ | \ | \ | Rabbit monoclonal | Cell signaling Technology |  |
| P38 /P-P38 | 1:500 | \ | \ | \ | Rabbit monoclonal | Cell signaling Technology |  |
| Erk1/2 /P-Erk1/2 | 1:400 | \ | \ | \ | Rabbit monoclonal | Cell signaling Technology |  |
| P-NF-κB (P65) | 1:500 | \ | \ | \ | Rabbit monoclonal | Cell signaling Technology |  |
| Ly6G | \ | 1:100 | 1:100 | \ | Rat monoclonal | BD |  |
| WB: Western Blot; IHC: Immunohistochemistry; IF: Immunofluorescence | | | | | | | |
