# Supplementary material for: Increased neutrophil extracellular traps promote metastasis potential of hepatocellular carcinoma via provoking tumorous inflammatory response
Source: J Hematol Oncol. 2020 Jan 6;13:3. doi: 10.1186/s13045-019-0836-0 (PMC6945602; doi:10.1186/s13045-019-0836-0)
Supplement: Supplementary file 2 — Additional file 2: Figure S1. NETs formation was enhanced in HCC associated neutrophils. Figure S2. Depositions of NETs was correlated with metastasis burden in HCC. Figure S3. NETs in HCC bearing mice. Figure S4. Evidence of NETs formation in TCGA and The Human Protein Atlas database. Figure S5. HCC enhanced the NETs formation capacity of normal neutrophils. Figure S6. NETs formation was enhanced in HCC-driven inflammatory condition in mice. Figure S7. Effect of DNase 1 and neutrophil depletion on LPS-induced NETs in mice. Figure S8. NETs trap optimized adhesion of HCC, and further promoted metastasis potential by raising invasion capacity with minimal cytotoxicity. Figure S9. Effect of DNase 1 on inflammatory mediators expression in NETs-stimulated HCC cells. Figure S10. Correlation analysis of infiltrated neutrophils and COX2 or other inflammatory mediator genes in TIMER database. Figure S11. Pearson correlation analysis of PADI4/MPO and COX2 or other inflammatory mediator genes in TCGA database. Figure S12. Effects of inhibiting IL-1 and TNF-α on NETs-aroused metastasis potential. Figure S13. HCQ impaired the enhanced metastasis potential triggered by NETs. Figure S14. Targeting NETs abrogated tumorous inflammatory response. [file 13045_2019_836_MOESM2_ESM.zip › Supplementary figures.doc]

**Figure S1. NETs formation was enhanced in HCC associated neutrophils. (A)** Typical morphological change of HCC neutrophils during NETs formation. NETs were detected by fluorescent immunostaining for DNA with Hoechst33342 and NETs components NE/H3cit/MPO. **(B)** Western blot analysis of H3Cit expression in the neutrophils from HD/HCC (n=4 each). **(C)** Western blot analysis of H3cit expression in the neutrophils from HCC tumor bearing C57BL/6 mice. **(D)** Extracellular NETs released by neutrophils from HCC tumor bearing C57BL/6 mice. Freshly isolated mice neutrophils with or without PMA stimulation were fixed and then stained for DNA with Hoechst33342. Symbol → indicated typical extracellular NETs structure. NETs-DNA quantification also seen in Figure 1C. Scar bar: 50μm.

**Figure S2. Depositions of NETs was correlated with metastasis burden in HCC.** **(A)** Detection of neutrophils (CD66b) in paraffin-embedded HCC samples (n=104). Neutrophils infiltrated mainly in tumor rather than paired non-tumor liver. No significant increasing trend of neutrophils infiltration was observed in metastasis/recurrence (n=16) than non-metastasis/recurrence (n=88) HCC samples. Scare bar: 100μm. **(B-C)** *In situ* immunofluorescence of NETs in paraffin-embedded HCC samples. NETs were identified by DNA, MPO, NE and/or H3cit, scare bar:100μm (B) or 20μm (C). *** *P*<0.001. Data were presented as means ± SEM.

**Figure S3. NETs in HCC bearing mice.** Analysis of NETs localization across HCC tissue from Hepa1-6 orthotopically implanted C57BL/6 mice. Scare bar: 50μm. ** *P*<0.01. Data were presented as means ± SEM

**Figure S4. Evidence of NETs formation in TCGA and The Human Protein Atlas database. (A) Pearson correlation analysis between PADI4 and CD66b(CEACAM8)/MPO in TCGA database. (B-C) Representative images and Pearson correlation analysis between PADI4 and MPO in The Human Protein Atlas database (https://www.proteinatlas.org).**

**Figure S5. HCC enhanced the NETs formation capacity of normal neutrophils**.

**(A)** Representative fluorescent images (left panel) of NETs formation as deposited DNA/MPO/NE in normal neutrophils incubated with HCC or HD plasma and quantification (right panel). Scare bar: 20μm. **(B)** Representative fluorescent images of NETs formation of neutrophils (lower chamber) in a co-culture system with HCC cell line (upper chamber). Neutrophils were fixed and then stained for chromatin (SytoxGreen). Symbol △ indicated representative extracellular NETs structure. Scare bar: 20 μm. **(C-D)** NETs formation from neutrophils incubated in CM of HCC cell lines. Normal neutrophils were pre-incubated with CM with or without additional PMA for 4 hours (C), or incubated with CM alone or CM plus PMA for 16 hours (D). Neutrophils were directly labeled with extracellular DNA dye SytoxGreen and total DNA dye Hoechst33342 during incubation to visualize NETs (left panel), and quantified for NETs-DNA (right panel). Scare bar: 20μm. Normal neutrophils were freshly isolated from peripheral blood of healthy donors. * *P*< 0.05; ** *P*< 0.01. Data were presented as means ±SEM.

**Figure S6. NETs formation was enhanced in HCC-driven inflammatory condition in mice. (A)** NETs formation of normal C57BL/6 mice neutrophils in a co-culture system with Hepa1-6 cells. Mice neutrophils (lower chamber) with NETs were fixed and then stained for chromatin and NE using immunofluorescence staining. Symbol → indicated representative extracellular NETs structure. Scare bar: 20μm. **(B)** Typical NETs formation of mice neutrophils pretreated with Hepa1-6 CM and followed by PMA stimulation (upper panel), or incubated with Hepa1-6 CM (lower panel). Neutrophils with NETs were fixed and stained for extracellular chromatin and NE using immunofluorescence staining. Symbol → indicated typical extracellular NETs structure. Scare bar:20μm.

**Figure S7. Effect of DNase 1 and neutrophil depletion on LPS-induced NETs in mice. C57/BL6 mice (n=4 each group) were given DNAse 1 (100U per mouse, intraperitoneal) or neutrophil-depleting antibody, and challenged by LPS as described in manuscript. Samples were collected 6 hours after LPS administration. (A) Sera MPO-DNA level in each group. (B) Typical images of NETs formation in situ by immunofluorescence staining and fluorescence signal analyze (ImageJ). Scare bar: 50 μm. * P<0.05, ** P<0.01, ns: not significant.**

**Figure S8. NETs trap optimized adhesion of HCC, and further promoted metastasis potential by raising invasion capacity with minimal cytotoxicity.** **(A)** Pretreatment with human NETs enhanced the adhesion of human HCC cells in a null-mice model, and this was again abrogated by DNase 1. Null-mice were subjected to NETs-producing human neutrophils stimulated with PMA with or without DNase 1 abrogation, and followed by Dil-labeled HepG2 cells through intraspleen/intravenous injection. Representative images of HCC cells adhesion in liver and lung under fluorescence microscope observation and quantification were shown. Scare bar:100μm. **(B)** Representative images of adhered Dil-labeled HepG2/MHCC97H cells *in vitro*. Quantification also seen in Figure 5C. Scare bar: 100μm. **(C)** Representative images of cytotoxicity on human HepG2/MHCC97H cells with NETs treatment plus (or not) DNase 1 abrogation as revealed by TUNEL assay. Quantification also seen in Figure 5D. Scare bar: 50 μm. **(D)** Little alteration of cytotoxicity on mice Hepa1-6 cells with NETs treatment or DNase 1 abrogation revealed by TUNEL assay. Representative images and quantification were shown. Scare bar: 50μm. **(E)** NETs enhanced invasion capacity of Hepa1-6 cells in a Transwell system, and this was abrogated by DNase 1. **(F)** Little alteration of *in vitro* proliferation capacity of mice Hepa1-6 cells with NETs treatment plus (or not) DNase 1 abrogation as revealed by CCK8 assay. **P*<0.05; ***P*<0.01. Data were presented as means ± SEM.

**Figure S9. Effect of DNase 1 on inflammatory mediators expression in NETs-stimulated HCC cells. Real-time PCR analyzing of inflammatory mediators expression in human HCC cell line HepG2 and MHCC97H 6 and 24 hours after NETs stimulation.**

**Figure S10. Correlation analysis of infiltrated neutrophils and COX2 or other inflammatory mediator genes in TIMER database.**

**Figure S11. Pearson correlation analysis of PADI4/MPO and COX2 or other inflammatory mediator genes in TCGA database.**

**Figure S12. Effects of inhibiting IL-1 and TNF-α on NETs-aroused metastasis potential. (A-B)** IL-1R antagonist Anakinra (100ng/mL) and TNF-α inhibitor Infliximab (10 μg/mL) exerted few effects on resistance to cytotoxic effect of NETs (A) and invasion capacity of cancer cells (B). Data were presented as means ±SEM.

**Figure S13. HCQ impaired the enhanced metastasis potential triggered by NETs.** The cytotoxicity and invasion capacity alteration in HepG2/MHCC97H/hepa1-6 cells pre-treated with HCQ and followed by NETs stimulation. *,#:*P*<0.05; **,##:*P*<0.01. Data were presented as means ± SEM.

**Figure S14. Targeting NETs abrogated tumorous inflammatory response.** The mRNA level change of tumorous COX2, IL-1β, IL-6, CXCL1/2, and TNF-α detected by RT-PCR. *:*P*<0.05; **:*P*<0.01. Data were presented as means ± SEM.
